# The rise and fall of religion: An agent-based model of secularisation, security and prosociality

**DOI:** 10.1371/journal.pone.0327674

**Published:** 2025-11-20

**Authors:** Ivan Puga-Gonzalez, F. LeRon Shults, Ross Gore, Konrad Talmont-Kaminski

**Affiliations:** 1 NORCE Center for Modelling Social Systems, Kristiansand, Norway; 2 Institute for Global Development and Planning, University of Agder, Kristiansand, Norway; 3 Center for Secure and Intelligent Critical Systems, Old Dominion University, Norfolk, Virginia, United States of America; 4 Society and Cognition Unit, University of Bialystok, Białystok, Poland; Beijing University of Posts and Telecommunications, CHINA

## Abstract

The relationship between religion, society, and individual behaviour has been a subject of extensive inquiry, drawing upon a rich collection of historical and contemporary perspectives. The scientific study of religion at the social level has often found its roots in the foundational work of Durkheim (Durkheim, 1912), who posited that religion serves as a catalyst for social order and the promotion of prosocial behaviour. At the same time, Malinowski’s observations regarding the connection between ritual and anxiety have led to a number of lines of inquiry that have come to extend to other aspects of religion. Yet, taken together, these two approaches create friction by simultaneously linking religion to low and high levels of environmental threats and anxiety. This becomes particularly relevant in discussions of secularisation in general and existential security in particular. This study embarks on a theoretical exploration of these approaches, connecting them through an agent-based computer simulation. By linking together some of the intricate mechanisms that underlie the dynamics of religion, prosociality, and anxiety, we aim to shed light on the conditions that give rise to highly religious societies and the subsequent decline in religiosity, with a view to the significance of central institutions that ensure cooperation without recourse to religion in this complex narrative.

## Introduction

Religion has been a pivotal force in the development of human societies and an integral part of every known large-scale society until recent times. Typically, research into religion’s effects has taken place on two distinct levels: the societal level, where religion’s role in fostering cooperation has been focused upon, and the individual level, where the causes and effects of personal religiosity are examined.

At the societal level, the scientific study of religion has usually drawn upon the work of Durkheim. Durkheim [1] characterised religion as primarily serving to create social order, to ensure that members of a community work together for their common good by promoting prosocial behaviour. This point has been explored by a range of studies [2–7] that have often shown that indeed, increased religiosity leads to a willingness to behave more altruistically towards the members of one’s ingroup. This hypothesis that religious beliefs and behaviours intensify or reinforce such “parochial” prosociality has been borne out by experimental studies in psychology, statistical analyses of survey data, and qualitative analysis of ethnographic and interview data [8–13].

At the individual level, Malinowski [14,15] observed that religious rituals become more common when people face situations that are dangerous and unpredictable – his example of Trobriander fishermen only engaging in rituals when going out to fish on the open ocean having been referred to countless times. Much work has followed, expanding Malinowski’s original observation to connect individual anxiety with a range of aspects of religion – including supernatural beliefs – at both individual and societal scales and over timescales ranging from that of minutes to that of years [16–22]. These studies illuminate the multifaceted nature of religious rituals and show that while religious rituals are indeed associated with anxiety at the individual level, they also have profound consequences at the group level, where they play a range of roles.

While both of these approaches have been highly influential, there has been a lack of discussion of the apparent friction between them. Given religiously-motivated cooperation and the resultant greater ability of religious societies to deal with threats, one would expect high levels of religiosity to correlate with lower levels of anxiety – which is exactly the opposite of what Malinowski suggested and what has often been observed. Furthermore, if religions are deemed indispensable to ensure cooperation, secularised societies should theoretically be chaotic rather than the peaceful and safe havens they actually are.

Recently, Talmont-Kaminski [23] has dealt with this contradiction by arguing that traditional societies are maintained by a prosocial equilibrium. He suggested a feedback loop between anxiety, religiosity, and prosocial behaviour that, outside of fundamental social changes, could lead to an equilibrium state with relatively high levels of social cooperation and religious engagement. The basic causal loop is one in which new external threats, that could negatively affect a society, lead to increased levels of anxiety among its members, thereby increasing their engagement with that society’s religious rituals and traditions. In turn, the strengthening of those religious traditions leads to their increased ability to promote prosocial behaviour, which makes the society more capable of countering the external threats and thereby maintains the society’s stability.

This conception of a religiously-motivated prosocial equilibrium abstracts away from other mechanisms that drive altruistic behaviour such as kin selection [24] and reciprocal altruism [25] because these mechanisms appear not to be sufficient to explain cooperation in large scale societies – precisely the societies that religion is thought to have played a significant role in helping to make possible. Once external threats are eliminated, anxiety can fall leading to a decrease in religiosity and cooperation. However, so long as external threats are sufficiently common and significant, religion and prosocial behaviour are maintained over time.

The feedback loop postulated by the prosocial equilibrium theory incorporates key insights from both Durkheim and Malinowski. The loop is closed by reference to the concept of credibility-enhancing displays (CREDS) developed by Henrich [26] where he explores how witnessing or participating in religiously-motivated behaviour (including prosocial behaviour) plays a vital role in maintaining religiosity, a claim that has received significant empirical support in recent work [27–29]. The religious institutions and practices that may emerge from this feedback loop serve as a kind of welfare system that fosters the growth of societies by offering individuals social support, protection, and health benefits by promoting prosocial behaviour and cooperation among in-group members. However, while the existence of a prosocial equilibrium provides an explanation for the rise and maintenance of religious societies, it does not by itself account for their decline, particularly noticeable in Western societies where the proportion of secular individuals and the decline of religious institutions is on the rise [30,31].

The phenomenon of secularisation has prompted various explanatory theories and factors such as increased educational attainment [32], religious pluralism [33], and existential security [34], etc. Notably, existential security theory explores the flip side of Malinowski’s insight at the societal scale. It suggests that secular institutions, such as the welfare state, might take over the role of religious institutions in ensuring cooperation and thereby providing basic needs, social and economic support, protection, etc., to the society. Over time, the functions of religious institutions become supplanted by secular counterparts, gradually diminishing the need for religiosity, and consequently, contributing to the observed decline of religiosity in many societies today [34]. Norris and Inglehart [34] have demonstrated these relationships across a wide range of countries and cultures, showing that religiosity remains strong in many developing societies (e.g., in Latin America, Middle East regions), is resurging in some post-Communist contexts (e.g., Eastern Europe), and is declining in most advanced industrial societies (e.g., western societies). The depth of change in religiosity varies across societies, as reflected in significant correlations between macro-level indices—such as the Human Development Index, societal modernization, and economic inequality—and religiosity. The underlying mechanism proposed by the prosocial equilibrium theory is that, by maintaining higher levels of security, secular institutions remove the drivers of religiously-motivated prosocial behaviour, thereby removing the means by which such credibility-enhancing displays of religion maintain high levels of religiosity in traditional societies.

In this study, we provide a conceptual and computational model of the prosocial equilibrium theory regarding the rise and decline of religiosity in societies. The purpose of this model is theoretical exposition [35]. Our objective is to comprehensively explore the theoretical assumptions of prosocial equilibrium theory and their implications by incorporating them into an agent-based computer simulation. We note that several other theories have been put forward to explain the process of secularisation, e.g., pluralism, education, freedom, secular competition, etc. [36]. The current study focusses upon existential security because of the relatively strong evidential support this theory already has [34,37].

Agent-based modelling has seen several notable studies exploring various aspects of religiosity. The functions of religious beliefs and ritual behaviours have been incorporated in a model simulating the emergence of mutually escalating intergroup conflicts [38]. Other models have demonstrated the role of education and existential security in the process of secularisation across several western cultures [11], and shed light on the mechanisms by which religious fundamentalism takes root in a society [39] and through which religious affiliation and disaffiliation persists at the regional level [40]. Still others have teased out the role of ritual form in the growth of religions [41] and the dynamics involved in “religious exiting” in secularizing contexts [33]. However, despite these valuable contributions, none of these models have undertaken the task of examining the specific theories we address here or providing a comprehensive theoretical exposition.

By examining the mechanisms hypothesized in prosocial equilibrium theory, and their interplay, we investigate how these factors interact and under what conditions they yield anticipated outcomes—namely, the growth of highly religious societies and subsequent declines in religiosity. Our aim encompasses two primary facets. Firstly, we revisit and extend an existing agent-based model by recreating previous findings that delve into the dynamics between religion, prosociality, and anxiety, and the emergence of growing religious societies [42]. In doing so, we explore the significance of the timing of reproduction, a variable previously left unexplored but with potential significant consequences on previous findings.

Secondly, and more significantly, we expand this model by introducing “central institutions” such as those that exist in many modern democracies which seek to protect members of a society against threats and are supported by universal contributions from all individuals regardless of their religious affiliation. This extension is particularly important given the context of existential security theory, as it sheds light on the plausibility of this highly influential theory. In light of these considerations, we explicitly set out the aims of the present study as follows:

1. To examine the theoretical assumptions of the prosocial equilibrium theory by recreating previous findings on the dynamics between religion, prosociality, and anxiety, and exploring the influence of reproduction timing on these dynamics.
2. To extend this model by incorporating central institutions, as informed by existential security theory, and evaluate the conditions under which such institutions contribute to the secularisation process.

## Methods

### Model conceptualization

**Fig 1:**
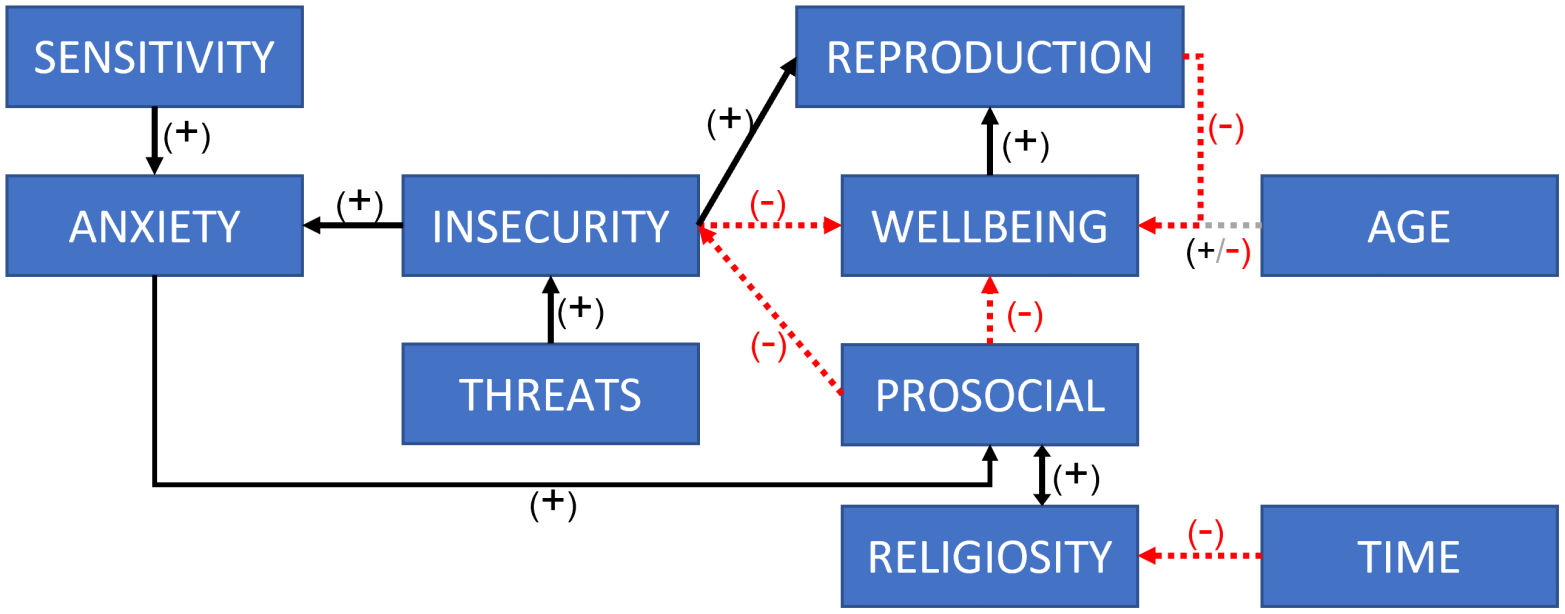
Model conceptualization. Red dashed lines represent negative effects and black lines represent positive effects. Central institution and its effects are depicted with a glow effect since they were introduced at the second stage (see Central Institutions section). For a full overview of the agents’ variables see Table 2.

We conceptualized our model as depicted in Fig 1. Our model attempts to implement the foundational claims about the relationships among three primary factors: religiosity, anxiety, and prosocial behaviour. Because of the ongoing discussions concerning the precise psychological and social mechanisms linking anxiety with religion and religion with cooperation, we have approached these elements with a degree of abstraction. These constructs are intended as simplified theoretical representations rather than empirically measured variables, consistent with the exploratory nature of the model.

To transform this theory into an agent-based model, we introduced several additional elements. In our model, threats represent external challenges or dangers that agents face, varying in frequency and intensity, and impacting their sense of security. This is an umbrella term representing a wide range of threats, from environmental (e.g., droughts, floods, earthquakes) to societal (e.g., wars, ethnic conflicts, overcrowding, socioeconomic instability). However, whether threats result in anxiety depends on sensitivity. Therefore, we incorporated insecurity and sensitivity as additional variables alongside anxiety. Where sensitivity refers to agents’ psychological vulnerability to experienced threats; insecurity measures the level of perceived instability and risk derived from experienced threats; and anxiety is the emotional response to agents’ insecurity via threats. Within this framework, anxiety emerges as a product of the multiplicative interaction between insecurity and sensitivity, reflecting their interconnected nature.

Prosocial behaviour is defined as behaviour that clearly incurs individual costs but benefits multiple others, such as spending time, energy, and resources to help build someone else’s house, taking care of elderly or ill people, sacrificing animals, mentoring, or tutoring others. Religiosity is an internal variable that is very closely connected to prosocial behaviour. On the one hand, whether one engages in prosocial behaviour depends upon the product of religiosity and anxiety. On the other, religiosity increases during the period of socialisation in response to witnessing or engaging in prosocial behaviour (a credibility-enhancing display). Given that prosocial behaviour carries costs, it negatively affects agents. Therefore, we incorporated a wellbeing variable representing physical health, living conditions, community engagement, economy, and other tangible aspects that may impact long-term the survival of the individual. Wellbeing is also negatively affected by insecurity. Most importantly, prosocial behaviour exerts a negative influence on insecurity, thereby indirectly reducing anxiety by removing its cause (see Fig 1). We assume that human societies deal with outside threats by cooperating (e.g., building a dyke to protect from flooding or catching a dangerous carnivore). This involves prosocial behaviours and in the model is represented as the negative effect of prosociality upon insecurity.

To address the conditions that lead to the growth of religious societies, we incorporated a reproduction mechanism. Agents reproduce when their wellbeing and insecurity levels are high, indicating that they prioritize reproduction in more insecure environments. While this may seem counterintuitive, this assumption finds support in existing literature: studies from Chicago neighborhoods found that high homicide rates, leading to lower life expectancy, correlated with earlier reproduction among women. This suggests that under high insecurity, prioritizing reproduction is an adaptive response to uncertain survival prospects [43]. Further, note that we purposefully omitted mechanisms that inherently favor the survival of religious societies. Although empirical data show an association between fertility and religiosity [44,45], including these mechanisms would have automatically made religious societies more likely to survive. Instead, our aim was to test whether religious societies can survive and thrive under equal reproductive conditions. Adding reproduction mechanisms specific to religious societies would have confounded our analysis.

We also included ‘age’ and ‘time’ to account for the evolution of societies over time. Age has a positive effect on wellbeing, but this effect eventually becomes negative as agents age. Time has a negative effect on religiosity, i.e., individuals decrease their religiosity every year from 12 to 25 years old. This is supported by research showing that adult religiosity is to a great degree determined by exposure to acts of religiosity during the socialisation years, 12–25, and that once adults, individuals are unlikely to lose or increase their acquired religious belief [30].

The complex interplay among all these variables is depicted in Fig 1. The equations governing the interrelationships within each of these processes are detailed in the subsequent sections. Also note that the central institution depicted in Fig 1 is introduced to the model in the second stage (see Central Institutions section for further details). A complete list and definitions of variables are provided in Table 1.

**Table 1:** Definitions of model conceptualization variables.

Variable	Definition
Age	The age of the individual agent
Anxiety	Emotional response to insecurity from threats (product of insecurity and sensitivity)
Central Institution	Entity protecting individuals from threats, regardless of religiosity
Insecurity	Perceived instability and risk from experienced threats
Prosocial behaviour	Costly actions benefiting others (e.g., caregiving, mentoring, almsgiving)
Religiosity	Internal variable set during socialisation and which promotes behaviour
Reproduction	Creation of new agents in the society
Sensitivity	Psychological vulnerability to threats
Threats	External dangers (e.g., natural disasters, wars, poverty)
Time	Time progression in the model representing real-world chronological time
Wellbeing	Physical, social and economic conditions affecting long-term individual survival

**Table 2:** Model parameters.

Parameter	Value	Description	Process
1. Rep Cost	CA	% of WB taken from each parent	Rep
2. Rep mid threshold	CA	Reproduction probability is 0.5	
3. Rep Curve Shape	CA	Parameters determining the shape of probability of reproduction curve	
4. Importance Insec	0.5		
5. Importance WB	1		
6. PB threshold	SA	Threshold value to trigger PB	PB
7. PB inc rel self	SA	Increase in agent’s and neighbours’ religiosity after a PB	
8. PB inc rel neigh	SA		
9. PB dec insec self	SA	Decrease in agent’s and neighbours’ insecurity after a PB	
10. PB dec insec neigh	SA		
11. PB wellbeing cost	SA	Decrease in agent’s WB after a PB	
12. Neigh Benefited	SA	# of nearby neighbours benefited	
13. Threats value	SA	Threat experienced every year	Threats
14. WB Age Threshold	CA	Parameters determining the increase/ decrease of WB according to agents’ age (see Wellbeing and Mortality processes)	WB-Age
15. WB Intercept C	CA		
16. WB Exp Gain eq	CA		
17. WB Exp Loss eq	CA		
18. WB Insec Threshold	0.1	Parameters determining the increase/ decrease of WB according to agents’ insecurity (see Wellbeing and Mortality processes)	WB-Insecurity
19. WB Max Inc	CA		
20. WB Max Dec	0.25		
21. Marriage Age Diff	CA	Max age difference between partners	Others
22. Radius Local Area	50	Radius of area of nearby neighbours	
23. Rel Dec Perc	SA	% of religiosity decrease every year	

WB = wellbeing; PB = Prosocial behaviour; Insec = insecurity; Rep = reproduction; inc = increase; dec = decrease; rel = religiosity; CA = calibrated parameter (see Reference Model section), SA = sensitivity analysis (see Simulations section).

### Model overview

The model is written in AnyLogic v.8.7.9. The model code, results, and R code to replicate the analyses are available at the following repository: https://gitlab.norceresearch.no/cmss/rip-project/-/tree/main/Prosociality%20subproject/Model%20with%20Central%20Institutions?ref_type=heads. Here we present a brief description, the full ODD + D protocol can be found as supporting information S2 Text.

The model simulates an artificial society initially inhabited by 1000 human agents. This number reflected a trade-off between computational efficiency and model stability. Given the stochastic nature of the model, simulations with smaller populations (e.g., 200–500 agents) exhibited a higher frequency of early extinctions, reducing the reliability of results. Substantially larger populations entail significantly increased runtime, limiting our ability to perform the number of replications necessary for robust statistical inference. Furthermore, while we did not systematically vary the population size, the data we have collected provides evidence that larger populations do not qualitatively alter the model’s dynamics. Agent behavior in our framework is governed by local, individual-level rules, and interactions are not network-mediated. Accordingly, increasing the number of agents would not introduce new interaction mechanisms. While the larger populations would dampen stochastic fluctuations and stability of the aggregate outcomes, the overall direction, structure, and qualitative nature of emergent patterns remain unchanged.

Each agent is characterized by eight variables: age, gender, marital status, religiosity, wellbeing, insecurity, sensitivity, and anxiety. These variables were deliberately chosen to keep the model as simple and tractable as possible, while still allowing us to explore the core mechanisms of interest—namely, how anxiety and religiosity interact to influence prosocial behaviour and societal survival. Age, gender, and marital status were included to enable a realistic representation of reproduction processes, which are essential for modeling demographic change.

On initialization, the agents’ age distribution is a pyramid shape (0–100 years), and their religiosity and sensitivity to external threats (sensitivity hereafter) are drawn from a normal distribution with a mean of 0.5 and standard deviation of 0.1. Insecurity is set to 0. These differences create a heterogeneous population, where agents vary in age, gender, marital status, religiosity, and sensitivity. This heterogeneity shapes not only their behaviour and outcomes, but also their wellbeing and in how they perceive and respond to their environment.

**Fig 2:**
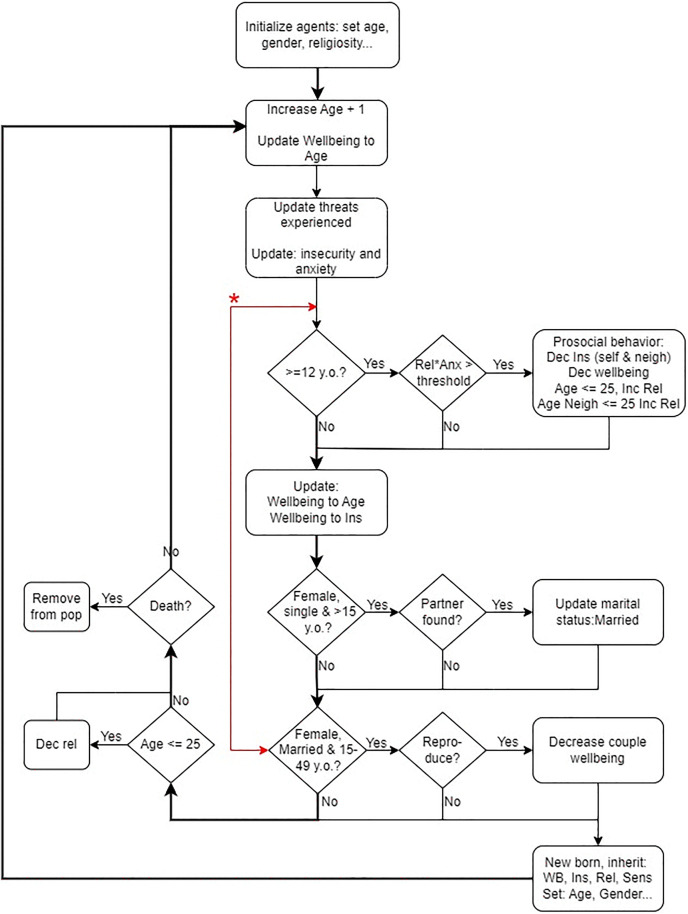
Model cycle and order of processes. *Depending on the setting, the opportunity for reproduction occurs (i) before, (ii) after, or (iii) randomly before/after prosocial behaviour.

Every year, the insecurity of agents increases due to external threats (13 in Table 2). If the insecurity exceeds 1, it is set to 1. The anxiety of an agent is the multiplicative effect of insecurity and sensitivity. Agents who are ≥ 12 years old are eligible to perform a prosocial behaviour (PB). PB occurs when the multiplicative interaction between anxiety and religiosity exceeds a certain threshold (6 in Table 2). Thus, PB is not a fixed state or attribute of the agent but rather an emergent action triggered under specific conditions. Performing PB reduces the insecurity of the performing agent and nearby neighbours (7–10 in Table 2) and increases their religiosity if they are 25 years old or younger but is costly and reduces the performing agent’s wellbeing (WB) (11 in Table 2). If there are more neighbours than the maximum number of individuals who can be benefited (12 in Table 2), the beneficiaries are selected randomly. WB increases or decreases according to the agents’ current age and insecurity values (14–20 in Table 2). Agents that are married, female, and within the reproductive age of 15–49 years old, have the opportunity for reproduction every year. Agents under 26 years of age reduce their religiosity annually by a set percentage (23 in Table 2). The probability of death is determined by the agents’ wellbeing value. The process flow diagram for the model is summarised in Fig 2.

### Wellbeing and mortality processes

Wellbeing (WB) determines the probability of an agent surviving every year. The survival probability curve mimics census data (1951–1955) from Norway. This choice was arbitrary, but it doesn’t have a major effect on the model’s behaviour. Both the reference model (see below) and the one with prosocial behaviour use the same survival probability curve, which means that because we compare one against the other the effect of the survival probability curve becomes irrelevant.

At initialization, WB is determined by a polynomial function of the agents’ age. This equation mimics the survival probability of both sexes according to age during the 1950s in Norway. After initialization, WB of agents increases and decreases every year according to their age. The gain or loss in WB is computed using two custom-designed equations that approximate typical curvilinear life-course dynamics and are not derived from existing empirical formulas. Other functional forms could have been used, but because both model variants use the same wellbeing dynamics, the specific choice does not affect the comparative results.

The gain in WB is given by equation 1:


$$Gain=-\text{4}C*{\left(\frac{Age-WB\_Age\_Threshold}{\text{1}\text{0}\text{0}-WB\_Age\_Threshold}\right)}^{Exp\text{1}}+C\;$$ (1)

The loss in WB is then given by equation 2:


$$Loss=-\text{4}C*{\left(\frac{Age-WB\_Age\_Threshold}{\text{1}\text{0}\text{0}-WB\_Age\_Threshold}\right)}^{Exp\text{2}}+C$$ (2)

Where *WB Age Threshold* (14 in Table 2) is the age at which the gain/loss in WB is given by equation 2 instead of equation 1; C (15 in Table 2) is the equation intercept, and *Exp1* and *Exp2* (16–17 in Table 2) determine the shape of the curve.

WB is also affected by the agent’s insecurity. Depending on the agent’s insecurity value and the value of *WB Insec Threshold* (18 in Table 2), wellbeing may increase or decrease every year according to equations 3 and 4 respectively.

If insecurity ≤ *WB Insec Threshold*:


$$Gain=WB.Max.Inc+\left(Ins*\frac{WB.Max.Inc}{WB.Insec.Th}\right)$$ (3)

*Ins* is the current insecurity of the agent, *WB.Max*.*Inc* represents the maximum gain in WB when insecurity equals 0, and *WB.Ins.Th* is the insecurity value at which there is neither gain nor loss in WB (18–19 in Table 2).

If *insecurity* > *WB Insec Threshold:*


$$Loss=\frac{-WB.Max.Dec}{\left(\text{1}-WB.Insec.Th\right)}*WB.Insec.Th+\left(Ins*\frac{WB.Max.Dec}{\left(\text{1}-WB.Insec.Th\right)}\right)$$ (4)

*Ins* is the current insecurity of the agent, *WB.Max.Dec* represents the maximum loss in WB when insecurity equals 1, and *WB.Ins.Th* is the insecurity value at which there is neither gain nor loss in WB (18–19 in Table 2).

The Gain and Loss functions in equations 1–4 are designed to mimic real-world patterns of wellbeing with age (or insecurity). This aligns with common understandings in the fields of psychology, health, and gerontology where a curvilinear relationship (e.g., an inverted U shape) is usually observed between wellbeing and age (or insecurity) [46]. The equations ensure that individuals experience increasing wellbeing up to a certain age (insecurity) threshold, after which wellbeing declines, reflecting common life experiences.

**Fig 3:**
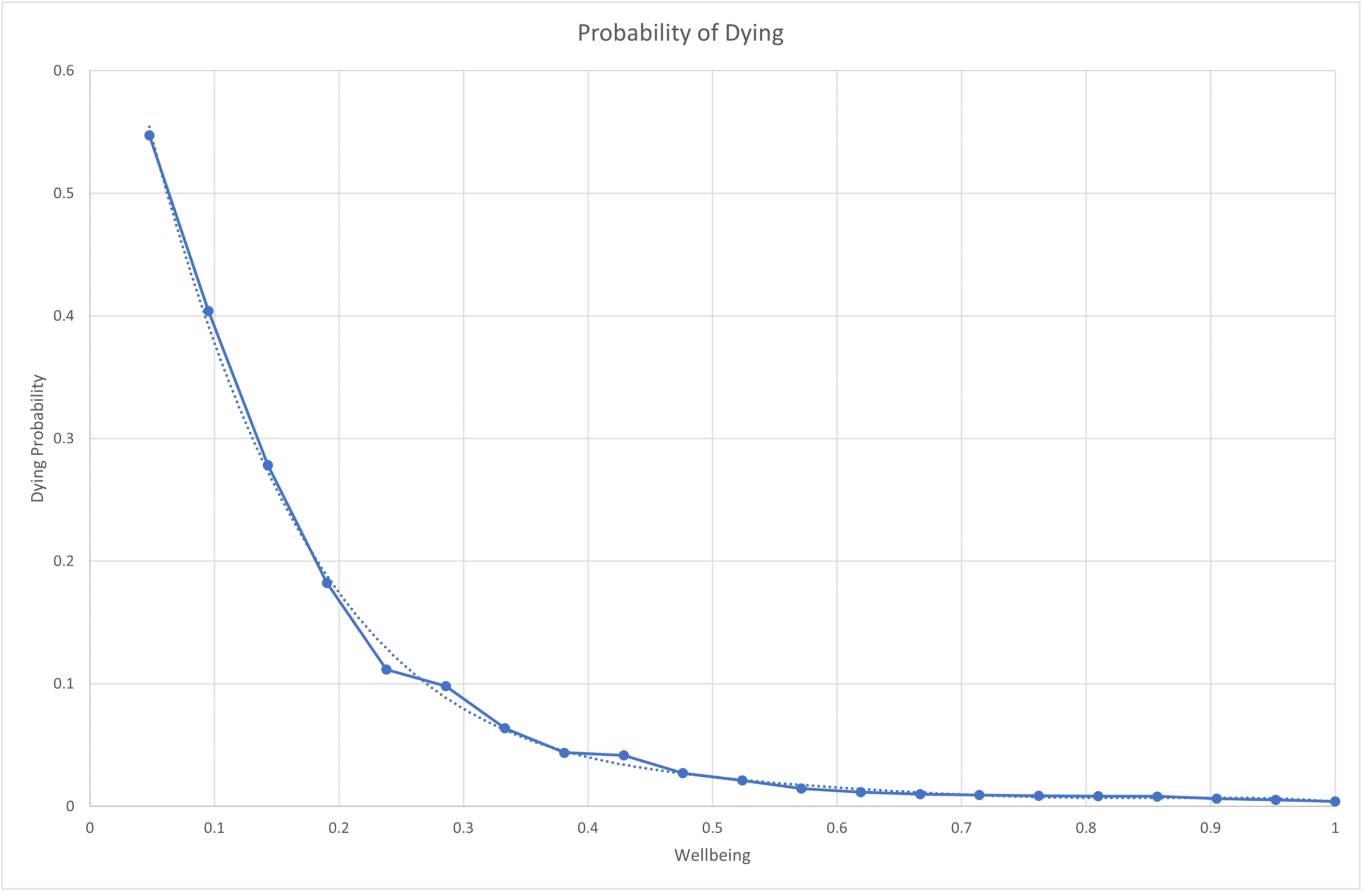
Probability of dying according to wellbeing.

WB determines the probability of agents dying and mimics the probability of dying in a particular year according to age reported in census data. To mimic this probability, we fitted a polynomial curve across census data (1951–1955) from Norway and input wellbeing instead of age. This resulted in the dying probability curve shown in Fig 3. Note that in the model, wellbeing functions as a distinct variable influencing death rates independently of *chronological* age. While there is a correlation between wellbeing and age, i.e., wellbeing tends to change as individuals age, it’s important to recognize that in the model, the relationship between wellbeing and the probability of death is not solely determined by age but also by insecurity and prosocial behaviours. This approach allows for a more nuanced representation of mortality risk recognizing that individual health and mortality risk can be influenced by factors beyond just age.

### Marriage and reproduction processes

Agents must meet three conditions to get married: (i) being single, (ii) being over 15 y.o., and (iii) having an age difference not higher than *Marriage Age Diff* between potential partners (21 in Table 2). If these conditions are met, an agent’s marital status is set to married. Once married, female agents in the age of reproduction [15–49] may reproduce every year. The probability of reproduction depends on the WB and insecurity of the married agents, and it is given by equation 5:


$$Prob.Rep=\frac{\text{1}}{\text{1}+e^{\left(-b*(x-a)\right)}}$$ (5)

Where *b* is the parameter *Rep curve shape* (3 in Table 2) determining the shape of the sigmoidal curve, *a* is the WB threshold at which reproduction probability is equal to 0.5 (2 in Table 2), and *x* is a weighted average of the partners’ WB and insecurity equal to:


$$\frac{(Average.WB)*Importance.WB+(Average.Ins)*Importance.Ins}{Importance.WB+Importance.Ins}$$ (6)

This weighted average represents the importance of WB and insecurity in the reproduction decision [43] (4–5 in Table 2). If agents reproduce, their WB is decreased by a percentage given by *Rep Cost* (1 in Table 2). The loss in WB from both partners is passed onto the offspring, and this value becomes the initial WB of the offspring. Offspring inherit the religiosity, insecurity, and sensitivity values from one of their parents (this parent is selected at random).

The timing of reproduction can occur (i) before PB, (ii) after PB, or (iii) at random, i.e., 50−50% chance before or after PB (Fig 2). We schedule reproduction at these different times because insecurity impacts the likelihood of reproduction: higher levels of insecurity result in a higher likelihood of reproduction and vice versa. Hence if reproduction occurs before PB, parents’ insecurity levels will be high, and reproduction likelihood will be higher. However, if reproduction occurs after PB, PB may have reduced parents’ insecurity and WB, potentially reducing or negating the likelihood of reproduction. To account for these variations, the timing of reproduction was scheduled as either before PB, after PB, or at random.

### Reference model

**Fig 4:**
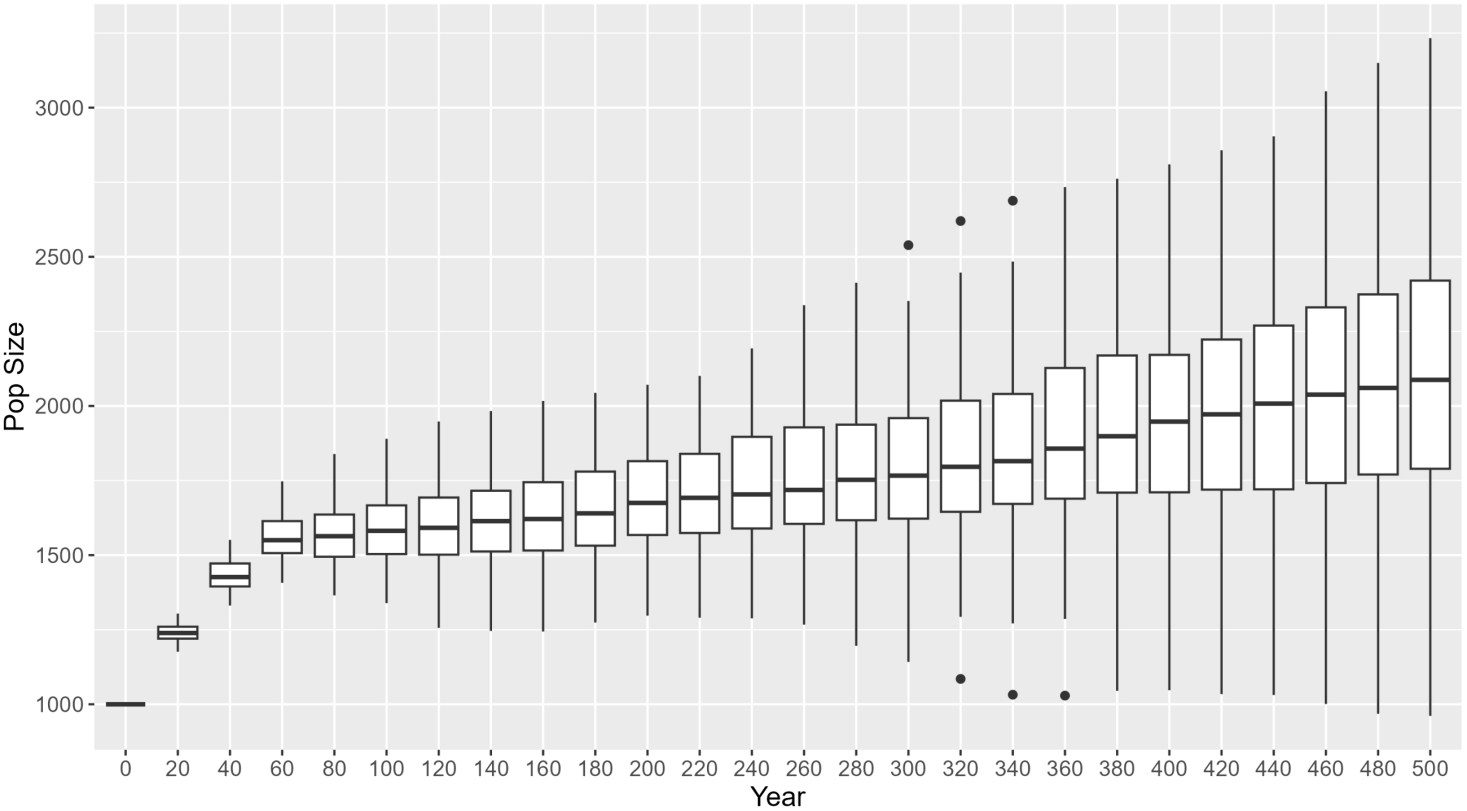
Reference model.

We created a reference model (RM) against which we could compare the effects of environmental threats and prosocial behaviour on the growth rate of society. The RM’s purpose is to establish a baseline for comparison and to determine if the lack of growth in a society is due to the society being unable to cope with environmental threats and/or insufficient prosocial behaviours or due to inappropriate parameter values for wellbeing, mortality, marriage, and reproduction processes. The RM was created by turning off environmental threats and prosocial behaviour and calibrating the parameters related to wellbeing, mortality, marriage, and reproduction (CA parameters in Table 2) to values that allow the society to maintain a slightly growing population over time. In the RM, the scheduling of reproduction has no effect since insecurity is always zero (no presence of threats of PB). Parameters were calibrated using the optimization engine in AnyLogic, which finds the combination of parameter values that maximizes or minimizes a specific output from an input function. The input function calculated the residual sum of squares between the observed yearly growth rate (pop_size_t+1_/pop_size_t_) and the expected growth rate if the population size remained constant over time, i.e., 1. The optimization experiments found the combination of parameter values that minimize the output value. We ran 10 optimization experiments, each ran for 500 time-steps (every time-step representing a year), and we chose the best one as the RM (Fig 4; see ODD + D protocol, S2 Text, for further details on the RMs).

### Simulations

To study the impact and validate the effect of threats on prosocial behaviour, religiosity, and society growth, we conducted a sensitivity analysis [47] by varying 9 parameters related to these factors (SA in Tables 2 and 3), and fixing the calibrated parameters of our reference model (RM). We used Latin-hypercube sampling to explore the parameter space 10,000 times (Table 3). These parameters were used to run the model under three different scenarios of reproduction: random, before, and after PB. For each set of parameters, we ran a simulation under each reproduction scenario, each lasting 600 time-steps (i.e., 600 years). We chose 600 years because threats and prosocial behaviour were introduced after the population reached stability at year 100 (Fig 4) and because we wanted to study the evolution of societies within a period of at least 500 years. We collected population size and average religiosity every 25 years. A society was considered successful if, at the end of the simulation, its population size exceeded 2500 individuals. We chose this value as it is greater than the median and the third quartile range of the reference model (RM) population size (Fig 4).

**Table 3:** Parameter space used in sensitivity analyses.

	1^st^ Analysis		2^nd^ Analysis		3^rd^ Analysis	
	MIN	MAX	MIN	MAX	MIN	MAX
1. PB threshold	0.001	0.500	0.001	***0.100***	0.001	***0.050***
2. PB inc rel self	0.100	0.500	0.100	0.500	0.100	0.500
3. PB inc rel neigh	0.100	0.500	0.100	0.500	0.100	0.500
4. PB dec insec self	0.100	0.500	0.100	0.500	***0.250***	0.500
5. PB dec insec neigh	0.100	0.500	0.100	0.500	***0.250***	0.500
6. PB wellbeing cost	0.001	0.500	0.001	***0.100***	0.001	***0.025***
7. Num Neigh Benefited	0.000	10.000	***5.000***	10.000	***5.000***	10.000
8. Threat value	0.001	0.500	0.001	0.500	0.001	0.500
9. Rel Dec Perc	0.001	0.500	0.001	0.500	0.001	0.500

Values that differ from the initial parameter space are in bold and italics (1^st^ Analysis). All parameters listed are SA parameters from Table 1. See “Simulations” section for further details.

## Results

**Fig 5:**
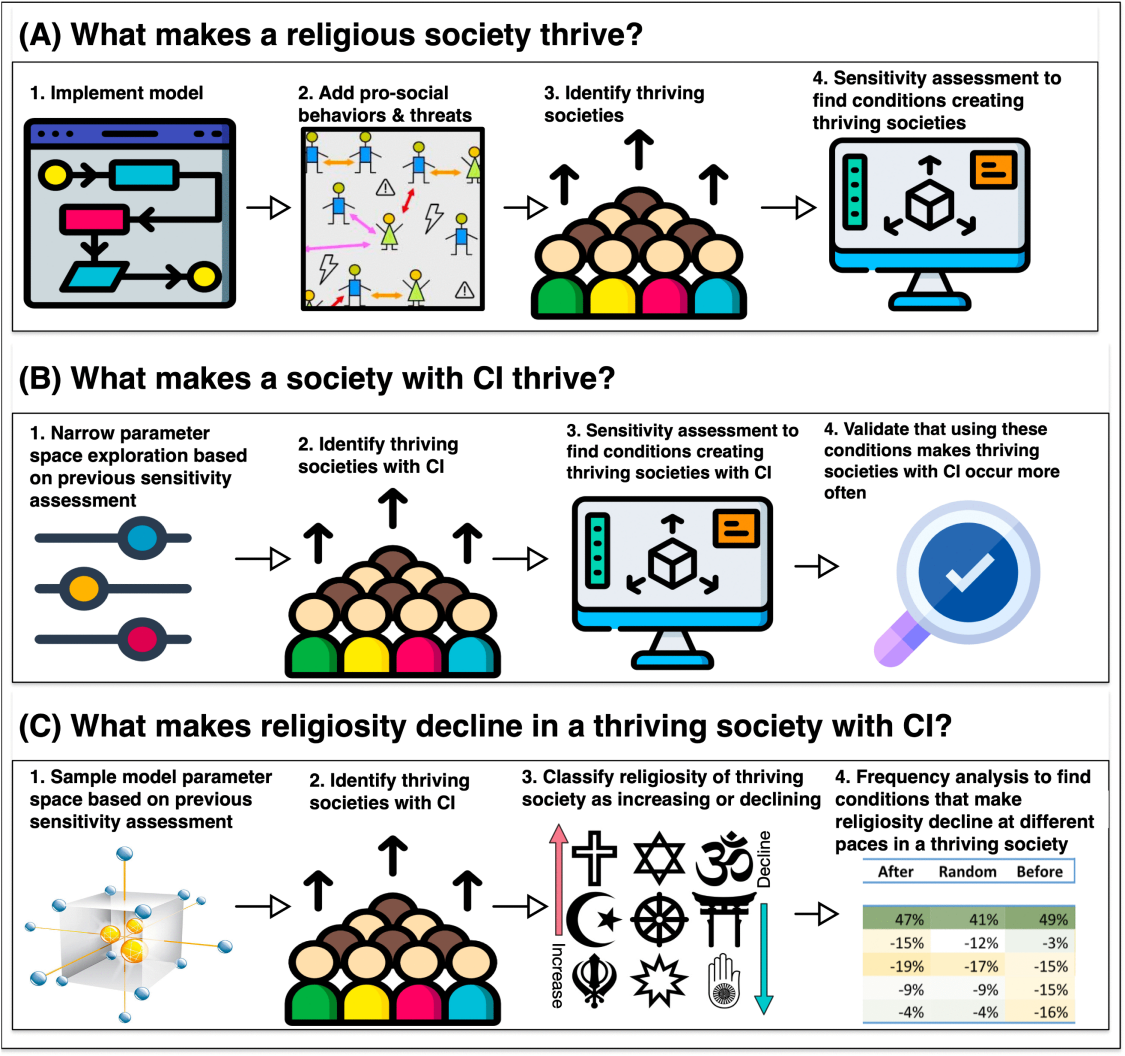
Roadmap to analyses.

Fig 5 provides a roadmap for our analyses and results. Initially, we identified the conditions conducive to the emergence of successful thriving societies (Fig 5A). Subsequently, we introduced central institutions (CI) into the model and further identified the conditions leading to successful thriving societies (Fig 5B). Finally, within the subset of successful thriving societies with CI, we pinpointed those exhibiting declining religiosity and the conditions leading to them (Fig 5C). The specifics of these results are elaborated below.

We used the Sensitivity Assessor Tool (whose use is illustrated in [48–50] and available at https://vmasc.shinyapps.io/SensitivityAssessor/), to identify and quantify the conditions that create successful societies in a model. A society was considered successful if its population size was greater than 2500 at year 600. Initial results showed that most societies (80%) became extinct before year 600, and only a very low percentage were successful (<0.04%), regardless of the three reproduction conditions (at random, before, after PB).

Therefore, we first focused on finding the conditions leading to surviving societies (pop sizes > 0). The sensitivity assessment identified three conditions that when present yielded surviving societies: (1) a PB threshold not greater than 0.1, (2) a PB wellbeing cost not greater than 0.1, and (3) a minimum of 5 neighbouring agents. The parameter space was then resampled with these new maximum and minimum values (Table 3; 2^nd^ Analysis), and led to higher percentages of successful societies: 22.95% for random reproduction, 21.98% for reproduction before PB, and 3.83% for reproduction after PB. Further analysis with the Sensitivity Assessor helped identify conditions leading to successful societies. The analysis suggested an even narrower parameter space for PB threshold, PB wellbeing cost, and decrease of insecurity on self and neighbours after PB (Table 3; 3^rd^ Analysis). Running simulations using these values resulted in even higher percentages of successful societies: 44.96% for random reproduction, 74.14% for reproduction before PB, and 24.12% for reproduction after PB.

**Fig 6:**
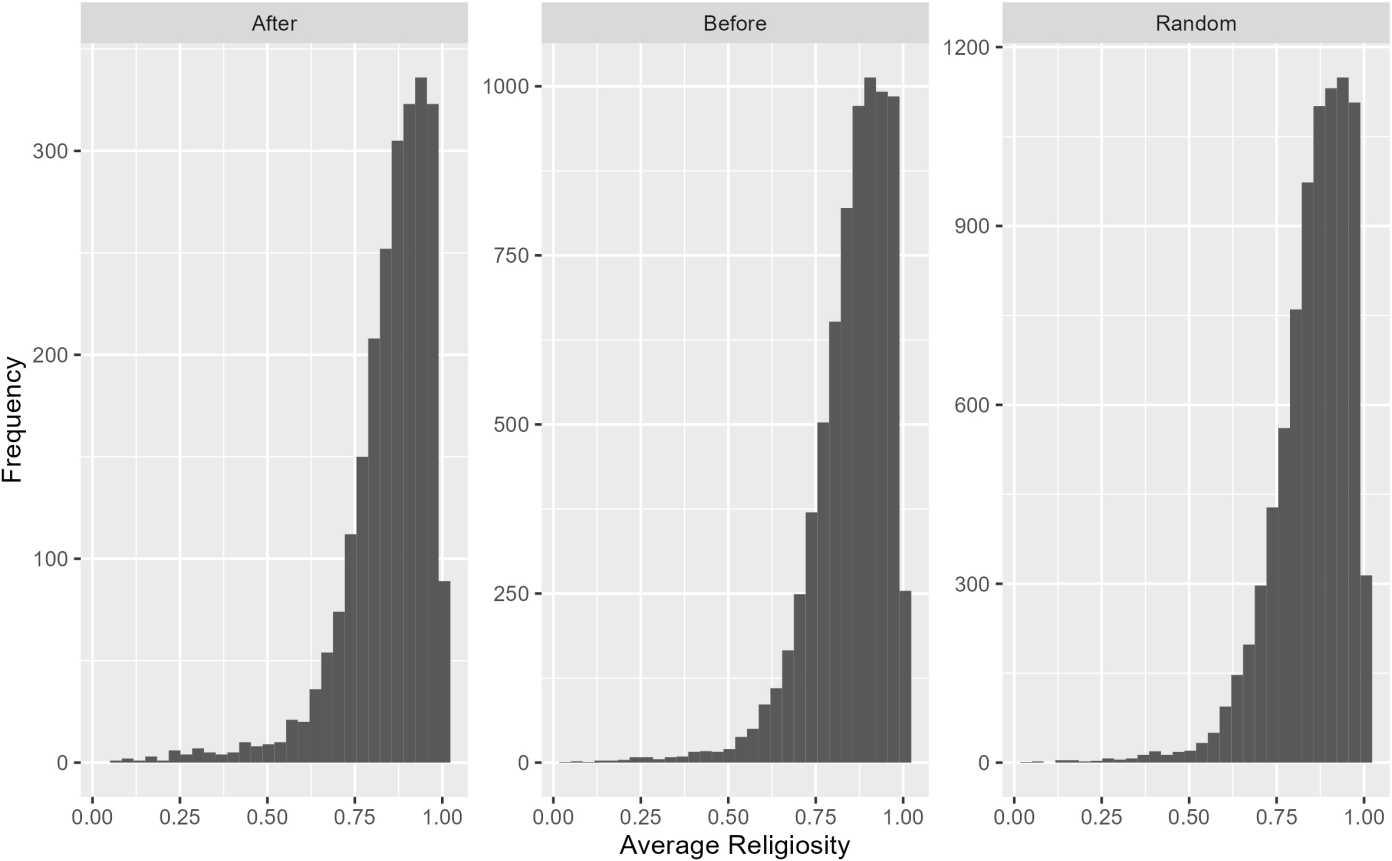
Average religiosity values of successful societies at year 600. Note that scales in the y-axes are different among the different reproduction times. This presentation highlights that the pattern is similar among the conditions despite different absolute values.

Our results demonstrate that the following four conditions are necessary for successful societies. First, the threshold of PB should be low, i.e., PB should be easily triggered in the face of threats. Second, PB should have a low cost for the performing agent. Third, the benefit of PB should be high, i.e., it should decrease insecurity of the performing agent and that of the benefited neighbours. Fourth, PB should benefit at least 5 agents other than the performing agent. It is also important to note that the logical ordering of reproduction significantly impacts the growth of societies with the most favourable being reproduction before PB. Identifying and quantifying the contributions of the reproduction order to successful societies demonstrates that: if reproduction occurs before PB, agents’ insecurity levels are presumably high, the probability of reproduction is thus also high, and societies grow faster. However, if reproduction occurs after PB, agents’ insecurity levels are presumably low, the reproduction output is therefore lower, and societies grow at a slower pace. Furthermore, religiosity plays a crucial role in successful societies, as most successful societies had an average religiosity value greater than 0.5 (95% of cases) or 0.75 (85% of cases) as shown in Fig 6. To grow, the great majority of societies need to maintain a high level of religiosity.

We also investigated the impact of stochastic threats and parochial prosociality on the emergence of successful societies. Parochial prosociality refers to behaviour where individuals only provide help to those who have a similar or higher level of religiosity. In the model, when this behaviour is activated, agents performing PB only benefit their neighbours who have a religiosity value higher than their own minus the parochial prosociality parameter value. The lower the value of the parochial prosociality parameter, the higher the required similarity in religiosity between the receiving neighbour and the performing agent to receive the PB benefit. The results of the simulations with stochastic threats were qualitatively the same as those with constant yearly threats. The percentage of societies with population sizes greater than 2500 was 31.80%, 79.80%, and 15.72% when the logical order of reproduction was random, before PB, and after PB, respectively. Parochial prosociality reduces the percentage of growing societies, as the benefit of PB is received by fewer agents (for detailed results of stochastic threats and parochial prosociality see S1 text).

### Central institutions

We explored the effect of Central Institutions (CI) on religiosity and population growth in the model. In the model, CI represent secular institutions that provide security to the population. When CI is activated, all agents are given a reduction in insecurity each year and in exchange, agents above 18 years old pay a cost in the form of a decrease in their wellbeing. This can be interpreted as analogous to a tax that everyone must pay regardless of (non)religiosity. However, this is only a rough analogy because our model is at a level of abstraction that does not include economic status or access to public goods as in some “club goods” models of religion [39]. The parameter space explored for CI is presented in Table 4. The parameters’ range remained unchanged from the third analysis (Table 3), except for the maximum values of threat and yearly decrease of religiosity, which were reduced to 0.3 and 0.25 respectively (Table 4). Further, in these simulations we included parochial prosociality, where a value of 1 brings us back to simulations without parochial prosociality (i.e., PB is given to neighbours no matter their religiosity value). Furthermore, the maximum cost of CI could be twice as much as the maximum PB cost and the minimum benefit of CI could be 2.5 times lower than the minimum PB benefit.

**Table 4:** Parameter space with central institution.

Parameter	MIN	MAX
PB threshold	0.001	0.050
PB inc rel self	0.100	0.500
PB inc rel neigh	0.100	0.500
PB dec insec self	0.250	0.500
PB dec insec neigh	0.250	0.500
PB wellbeing cost	0.001	0.025
Num Neigh Benefited	5.000	10.000
Threat value	0.001	0.300
Rel Dec Perc	0.001	0.250
Parochial Prosociality	0.200	1.000
**CI WB cost**	**0.001**	**0.050**
**CI Benefit**	**0.100**	**0.500**

In bold the two parameters related to Central Institutions.

**Fig 7:**
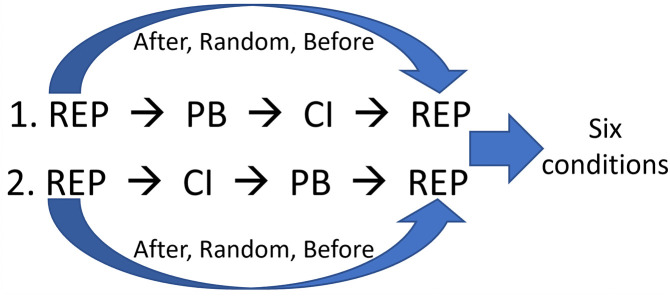
Different schedules of events tried out with CI. REP, reproduction; PB, prosocial behaviour; CI, central institution.

As with reproduction, the timing of the effect of CI in the model cycle can affect the results. Therefore, simulations were run with the CI scheduled both before and after PB, resulting in six combinations (Fig 7). Only the results where the CI effect occurs after PB are shown here, as the results are the same regardless of the timing. The effect of CI started in year 200, after societies had experienced 100 years of threats and religiosity-driven prosocial behaviours. The parameter space was sampled 5000 times and used to run the model for 600 years under the six conditions.

Societies were categorized as dying, surviving, or thriving based on a Pearson correlation between population size and year, starting at year 200. If the correlation was significant and negative (e.g., ‘- ‘) (p<0.1), the society was classified as dying, if non-significant it was classified as surviving, and if significant and positive (e.g., ‘+’) (p < 0.1), it was classified as thriving. Table 5 displays the percentage of societies in each category when the CI effect occurs after PB and the reproduction event occurs after, randomly, or before PB. Table 5 demonstrates that most societies either survive or thrive, particularly when reproduction occurs before PB. The average religiosity of societies in year 600 follows a bimodal distribution in all societal categories and time schedules of reproduction, with most societies having high religiosity (close to 1), but also a significant number having lowest religiosity (0), and few societies in between (Figure 2 in S1 Text).

**Table 5:** Percentage of societal category when CI institution effect occurs after PB and reproduction occurs after, at random, or before PB.

	After	Random	Before
Dying	48.92%	44.24%	7.02%
Surviving	9.46%	6.76%	3.82%
Thriving	41.62%	49.00%	89.16%

As our focus is on societies where CI helps them grow, we only analysed thriving societies. Thriving societies were further classified based on the correlation between average religiosity and year, starting at year 200. If the Pearson correlation was significant (p < 0.1) and negative, the society was classified as having declining religiosity, if non-significant (p > 0.1) it was classified as having stable religiosity, and if significant (p < 0.1) and positive, it was classified as having increasing religiosity. Table 6 shows the proportions of societies with declining, increasing, and stable religiosity from year 200 to year 600. Most thriving societies continue to increase their religiosity, but approximately 25% of them have declining religiosity. It is on those thriving societies with declining religiosity (TSDR hereafter) that we focus next.

**Table 6:** Proportions of thriving societies with declining, increasing, and stable religiosity according to the different time schedules of reproduction.

		Declining religiosity	Increasing religiosity	Stable religiosity
Reproduction schedule	After	24.10%	70.40%	5.48%
	Random	26.20%	64.10%	9.67%
	Before	15.50%	79.80%	4.76%

We observed that the rate of decline in religiosity varies among TSDR. To better understand this, we divided the TSDR societies into three categories based on the pace of decline in religiosity: slow/medium/fast decline, medium/fast decline, and fast decline (Figure 3 in S1 Text). The division was made as follows: the first category included all societies with a negative Pearson correlation between years 200–600, the second and third categories included societies with a negative Pearson correlation and an average religiosity of less than 0.5 and 0.125 at year 600, respectively. Using the Sensitivity Assessor Tool, we then investigated the conditions that led to a decline in religiosity in each of these three categories.

Table 7 shows the conditions identified by the Sensitivity Assessor. To identify these conditions, for each parameter we divided its parameter space into specific range values. We then measured the percentage of TSDR that fell within that specific range, i.e., the observed percentage. We also measured the percentage of all thriving societies that fell within that same range, i.e., the expected percentage. This strategy has been used by computer scientists to identify bugs more effectively in software where there are large differences in the number of test cases that meet the software requirements versus the number of test cases that fail to meet requirements [51,52]. Here we apply it to identify and quantify conditions contributing to declining religiosity in societies.

**Table 7:** Percentage difference (Observed – Expected) of societies with declining religiosity at different paces and within a specific parameter range.

	Declining Rel S/M/F			Declining Rel M/F			Declining Rel F		
	After	Random	Before	After	Random	Before	After	Random	Before
**1) Yearly threat**									
[0.001 - 0.06]	47%	41%	49%	63%	60%	70%	64%	65%	76%
(0.06 - 0.12]	−15%	−12%	−3%	−26%	−25%	−12%	−27%	−28%	−15%
(0.12 - 0.18]	−19%	−17%	−15%	−22%	−21%	−20%	−22%	−22%	−21%
(0.18 - 0.24]	−9%	−9%	−15%	−10%	−10%	−19%	−10%	−11%	−20%
(0.24 - 0.30]	−4%	−4%	−16%	−4%	−4%	−19%	−4%	−4%	−20%
**2) PP**									
[0.20 - 0.36]	8%	12%	17%	5%	6%	5%	3%	3%	2%
(0.36 - 0.52]	−2%	−1%	−3%	−5%	−2%	−2%	−4%	−2%	−4%
(0.52 - 0.68]	−1%	0%	−3%	3%	0%	1%	3%	1%	2%
(0.68 - 0.84]	−3%	−5%	−5%	−2%	−3%	−2%	−1%	−1%	0%
(0.84 - 1.00]	−3%	−5%	−6%	−1%	−2%	−2%	−1%	0%	0%
**3) PB threshold**									
[0.001 - 0.01]	−12%	−12%	−13%	−14%	−15%	−15%	−14%	−16%	−15%
(0.01 - 0.02]	−4%	−5%	−5%	−5%	−4%	−6%	−5%	−5%	−6%
(0.02 - 0.03]	2%	3%	1%	2%	0%	3%	2%	2%	3%
(0.03 - 0.04]	5%	6%	6%	7%	9%	7%	6%	7%	7%
(0.04 - 0.05]	9%	8%	11%	9%	10%	11%	10%	12%	12%
**4) PB WB cost**									
[0.001 - 0.005]	−3%	−4%	1%	−2%	−2%	3%	−1%	−1%	3%
(0.005 - 0.010]	−5%	−2%	−3%	−9%	−7%	−7%	−10%	−9%	−8%
(0.010 - 0.015]	−2%	−1%	−1%	0%	1%	0%	−1%	1%	1%
(0.015 - 0.020]	3%	2%	1%	3%	2%	1%	4%	2%	1%
(0.020 - 0.025]	8%	4%	2%	9%	5%	3%	8%	6%	3%
**5) CI benefit**									
[0.10 - 0.18]	−7%	−4%	−8%	−12%	−9%	−13%	−12%	−9%	−14%
(0.18 - 0.26]	−2%	−2%	−3%	−3%	−3%	−3%	−4%	−4%	−4%
(0.26 - 0.34]	3%	1%	2%	0%	2%	3%	0%	1%	3%
(0.34 - 0.42]	3%	3%	5%	6%	5%	5%	6%	6%	6%
(0.42 - 0.50]	3%	2%	4%	8%	6%	9%	10%	6%	9%
**6) CI WB cost**									
[0.001 - 0.01]	−1%	−1%	0%	−3%	−5%	1%	−3%	−4%	2%
(0.01 - 0.02]	−4%	−4%	−1%	−2%	−3%	−1%	−4%	−4%	−2%
(0.02 - 0.03]	−1%	1%	1%	−2%	0%	−1%	−2%	−1%	−1%
(0.03 - 0.04]	1%	1%	0%	0%	3%	0%	1%	3%	0%
(0.04 - 0.05]	4%	4%	−1%	8%	5%	1%	8%	6%	1%
**7) Rel Dec Perc**									
[0.001 - 0.05]	0%	−3%	−4%	1%	−3%	−2%	1%	−2%	−1%
(0.05 - 0.10]	−4%	−5%	−6%	−3%	−4%	−3%	−1%	−1%	−1%
(0.10 - 0.15]	0%	0%	−3%	2%	2%	−1%	2%	2%	−1%
(0.15 - 0.20]	2%	2%	5%	0%	3%	3%	0%	1%	3%
(0.20 - 0.25]	1%	6%	8%	−1%	2%	3%	−2%	0%	−1%

Colour scale goes from dark yellow (negative) to dark green (positive). Positive (dark green) values mean that more TSDR were present than expected and vice versa for negative (dark yellow) values. Note that the colour scale is adjusted to the percentage range within each parameter.

The percentages in Table 7 are the difference between the observed and expected percentage. A positive value indicates that more TSDR were observed in that range than expected, meaning this parameter range favours the occurrence of TSDR, and vice versa. The yearly increase in threat has the major effect in declining religiosity in thriving societies, low values (0.001–0.06) favour the occurrence of TSDR, while larger values (>0.06) counter it (Table 7). This suggests that CI can handle threats up to a certain threshold, and PB is needed for higher threats. This effect, however, could be exacerbated by the value range of CI parameters, allowing for larger benefits and lower costs of CI may increase the yearly threat range favouring TSDR.

Low values of parochial prosociality (PP) also support the occurrence of TSDR, but to a lesser extent than yearly threat, and this effect decreases as the pace of declining religiosity accelerates. Low PP values restrict the PB benefit to those with similar religiosity, resulting in fewer neighbours receiving the benefit, causing overall religiosity to decline as religiosity is not reinforced among those without the benefit.

When it comes to the remaining parameters in Table 7 (3–7), low values of the PB threshold parameter counteract the occurrence of TSDR because, in these cases, PB is easily triggered even at low threat levels, which helps to prevent the decline of religiosity. On the other hand, at high PB threshold values, PB is rarely triggered, and religiosity is not reinforced. This effect is more pronounced in societies with a fast pace of declining religiosity. Additionally, low costs of PB counter the occurrence of TSDR, while high costs favour it; and this effect appear equals across the different paces of declining religiosity. The benefit provided by CI also plays a role, with low benefits countering the occurrence of TSDR and high benefits favouring them. This effect is exacerbated in societies with a fast-declining pace. What is surprising, however, is that when the cost of CI is low, the occurrence of TSDR is countered, while high costs favour them. Finally, the yearly decrease in religiosity appears to have an impact on societies with a slow or medium decline pace, with low values countering the occurrence of TSDR, while greater ones favour them.

To evaluate the relative importance of each parameter in relation to others, we analysed conditions in which we combined the ranges of two parameters (Table 8). The lower and upper halves refer to the range values of each parameter. According to Table 8, PB threshold and yearly threat are the two most significant parameters affecting the occurrence of TSDR. Even when combined with other parameters, their main effect is reduced but not eliminated. As shown in Table 7, low values of PB threshold counteract TSDR, and this is also observed in all combinations of PB threshold values and other parameter values (1–6 in Table 8). When PB threshold values are in the lower half of the range, the percentage difference is always negative, indicating that TSDR is countered, and vice versa when PB threshold values are in the upper half, the percentage is positive, favouring the occurrence of TSDR. Therefore, the effect of PB threshold remains regardless of the value of the other parameter. The same holds true for yearly threat, except when combined with PB threshold (1 in Table 8). In all other cases (7–11 in Table 8), when yearly threat values are in the lower half, TSDR is favoured and vice versa, regardless of the value of the other parameter. Thus, PB threshold and yearly threat seem to play a crucial role in determining the occurrence of TSDR. To see the effect of other combination of parameters see Table 5 in S1 Text.

**Table 8:** Percentage difference (Observed – Expected) of societies with declining religiosity at different paces and within a specific combination of parameters range.

	Declining Rel S/M/F			Declining Rel M/F			Declining Rel F		
CONDITIONS	After	Random	Before	After	Random	Before	After	Random	Before
**1) PB threshold AND Yearly Threat**									
Lower half AND Lower half	−6%	−6%	3%	−8%	−9%	3%	−8%	−9%	3%
Lower half AND Upper half	−10%	−10%	−21%	−12%	−13%	−25%	−12%	−13%	−25%
Upper half AND Lower half	27%	26%	36%	33%	33%	45%	32%	34%	48%
Upper half AND Upper half	−11%	−10%	−18%	−13%	−11%	−23%	−13%	−12%	−25%
**2) PB threshold AND PB WB cost**									
Lower half AND Lower half	−13%	−11%	−11%	−18%	−17%	−15%	−19%	−18%	−16%
Lower half AND Upper half	−3%	−5%	−7%	−2%	−4%	−7%	−1%	−4%	−6%
Upper half AND Lower half	3%	5%	8%	5%	8%	10%	5%	8%	9%
Upper half AND Upper half	13%	11%	10%	15%	14%	12%	15%	14%	13%
**3) PB threshold AND Rel Dec Perc**									
Lower half AND Lower half	−11%	−13%	−15%	−10%	−13%	−12%	−10%	−11%	−11%
Lower half AND Upper half	−5%	−3%	−3%	−10%	−9%	−10%	−10%	−11%	−12%
Upper half AND Lower half	8%	5%	4%	11%	7%	8%	12%	9%	9%
Upper half AND Upper half	8%	11%	14%	9%	15%	14%	8%	13%	13%
**4) PB threshold AND PP**									
Lower half AND Lower half	−1%	0%	−2%	−6%	−7%	−8%	−7%	−9%	−10%
Lower half AND Upper half	−15%	−16%	−16%	−14%	−15%	−14%	−13%	−13%	−12%
Upper half AND Lower half	9%	11%	15%	8%	11%	12%	8%	10%	9%
Upper half AND Upper half	7%	5%	3%	12%	11%	10%	12%	12%	13%
**5) PB threshold AND CI WB cost**									
Lower half AND Lower half	−13%	−11%	−9%	−15%	−15%	−11%	−15%	−15%	−12%
Lower half AND Upper half	−4%	−5%	−9%	−5%	−7%	−11%	−4%	−7%	−11%
Upper half AND Lower half	7%	6%	9%	8%	6%	11%	7%	7%	11%
Upper half AND Upper half	9%	10%	9%	12%	15%	11%	13%	16%	11%
**6) PB threshold AND CI benefit**									
Lower half AND Lower half	−9%	−8%	−11%	−10%	−9%	−12%	−11%	−10%	−13%
Lower half AND Upper half	−7%	−8%	−7%	−10%	−12%	−10%	−9%	−12%	−9%
Upper half AND Lower half	2%	4%	2%	−4%	0%	−4%	−5%	0%	−3%
Upper half AND Upper half	14%	13%	16%	24%	22%	26%	25%	23%	25%
**7) Yearly threat AND PB WB cost**									
Lower half AND Lower half	5%	8%	18%	4%	6%	20%	3%	6%	20%
Lower half AND Upper half	16%	12%	21%	21%	17%	28%	22%	18%	30%
Upper half AND Lower half	−15%	−14%	−21%	−17%	−16%	−26%	−17%	−17%	−27%
Upper half AND Upper half	−6%	−6%	−18%	−7%	−8%	−23%	−7%	−8%	−24%
**8) Yearly threat AND Rel Dec Perc**									
Lower half AND Lower half	9%	4%	14%	13%	6%	20%	14%	11%	24%
Lower half AND Upper half	12%	16%	25%	12%	17%	28%	10%	14%	27%
Upper half AND Lower half	−12%	−12%	−25%	−12%	−13%	−25%	−12%	−13%	−25%
Upper half AND Upper half	−9%	−8%	−14%	−12%	−11%	−24%	−12%	−12%	−26%
**9) Yearly threat AND PP**									
Lower half AND Lower half	17%	18%	27%	14%	14%	28%	13%	13%	25%
Lower half AND Upper half	5%	2%	12%	10%	9%	21%	11%	12%	25%
Upper half AND Lower half	−9%	−7%	−14%	−12%	−11%	−23%	−12%	−12%	−25%
Upper half AND Upper half	−12%	−13%	−25%	−12%	−13%	−25%	−12%	−13%	−25%
**10) Yearly threat AND CI WB cost**									
Lower half AND Lower half	11%	11%	19%	13%	10%	25%	11%	11%	26%
Lower half AND Upper half	10%	10%	20%	11%	13%	23%	13%	14%	24%
Upper half AND Lower half	−17%	−15%	−20%	−19%	−19%	−26%	−19%	−19%	−27%
Upper half AND Upper half	−4%	−5%	−19%	−5%	−5%	−23%	−5%	−5%	−23%
**11) Yearly threat AND CI benefit**									
Lower half AND Lower half	0%	1%	12%	−7%	−4%	7%	−8%	−4%	7%
Lower half AND Upper half	21%	20%	27%	31%	28%	42%	32%	29%	44%
Upper half AND Lower half	−7%	−5%	−21%	−7%	−6%	−23%	−7%	−6%	−23%
Upper half AND Upper half	−14%	−15%	−18%	−17%	−18%	−26%	−17%	−19%	−28%

Colour scale goes from dark yellow (negative) to dark green (positive). Positive (dark green) values mean that more TSDR were present than expected and vice versa for negative (dark yellow) values. Note that the colour scale is adjusted to the percentage range within each combination of parameters.

The results of our analyses so far identified five parameters that play a crucial role in the emergence of TSDR: PB threshold, threat intensity, yearly religiosity decrease, parochial prosociality, and CI benefit. To further study the impact of these parameters on the religiosity of thriving societies, we performed another analysis where we varied each of these five parameters by a specific value while keeping all other values constant (see Table 6 in S1 text). The resulting 10,800 combinations of parameters (5 x 10 x 6 x 6 x 6) were run under three different timings of reproduction conditions: before PB, at random, or after PB.

Table 9 displays the percentage of societies dying, surviving, and thriving resulting from the new parameter space. The results showed that compared to Table 5, there was an increase in the percentage of thriving societies when reproduction occurred after or at random. In addition, among the thriving societies, most of them had declining religiosity, as shown in Table 10.

**Table 9:** Percentage of societies in each condition according to timing of reproduction.

	After	Random	Before
Dying	15.6%	11.8%	5.8%
Surviving	11.1%	7.3%	5.4%
Thriving	73.3%	80.9%	88.8%

Simulations were run using the parameter values shown in Table 6 in S1 Text.

**Table 10:** Religiosity trend among thriving societies.

		Declining religiosity	Increasing religiosity	Stable religiosity
Reproduction schedule	After	52.0%	25.5%	22.5%
	Random	52.4%	23.7%	23.9%
	Before	47.9%	32.1%	20.0%

Simulations were run using the parameter values shown in Table 6 in S1 Text.

**Fig 8:**
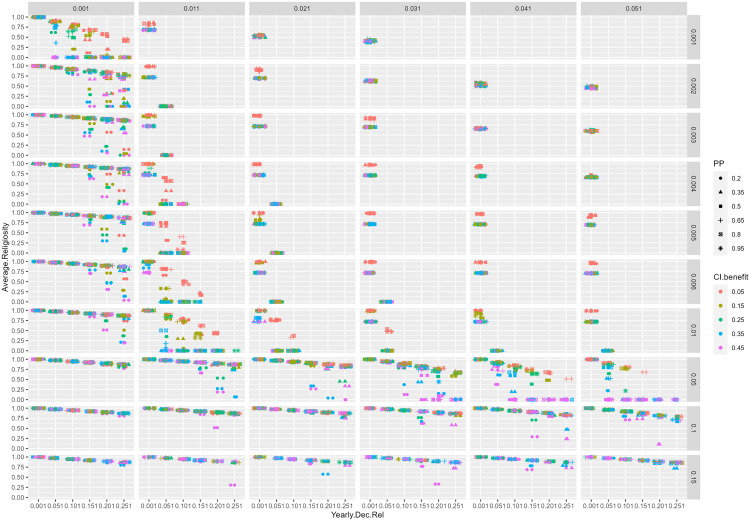
Average religiosity of thriving societies according to different parameters’ values and when reproduction occurs after PB. Data points are jittered along the x-axis for best visualization.

The results of these simulations for thriving societies are presented in Fig 8 (for results with reproduction occurring at random and before PB see Figures 4 and 5 in S1 Text). Each figure is a six-dimensional graph with the following elements:

1. The x-facet shows the values of the PB threshold.
2. The y-facet displays the yearly threat intensity.
3. The x-axis represents the yearly decrease in religiosity.
4. The y-axis shows the average religiosity at year 600.
5. The colour of the data points represents the CI benefit.
6. The shape of the data points displays the degree of PP, with lower values indicating a higher degree of parochialism.

Based on these results, several observations can be made:

- If religion decreases slowly every year (0.001 on the x-axis), societies never lose their religiosity, regardless of the other parameters in the model. This result lends plausibility to the claim that secularisation hinges on a delicate equilibrium between forgetting religious beliefs (yearly religiosity decay parameter) and reinforcing them through prosocial behavior (PB). Without a minimal level of forgetting, societies are unlikely to undergo secularisation. This aligns with other research suggesting that in secular societies religious beliefs are somewhat naturally forgotten because they are not actively reinforced over time [53,54].
- When threats are very high (two bottom rows of the y-facet), religiosity remains high except in a few cases where the CI benefit (0.35–0.45, blue/purple colours), PP (0.2–0.35, circle/triangle shapes), and yearly decrease of religiosity (0.151–0.251, x-axis) are medium-high. This suggests that religiosity persists in societies where threats are intense and frequent. Under such conditions, even if the benefit of CI is high and parochial prosociality is also high, religiosity still only decreases up to a certain point but does not disappear. Such output scenarios resemble environments characterized by ecological, social or other stressors in the real world, where parochial prosociality plays a significant role [38,55,56].
- If threats are low to medium (first 7 top rows in the y-facet) and PB is not easily triggered (>0.021, x-facet), thriving societies do not exist unless the yearly decrease in religiosity is slow (<0.051, x-axis). This suggests a fine balance between experiencing threats, the threshold needed to trigger a PB (governed by anxiety and religiosity levels), and forgetting about religious beliefs. Remember that PB is triggered when the multiplicative interaction between anxiety and religiosity exceeds a certain threshold. Hence, if threats are not strong enough to elicit PB (via insecurity and anxiety), and religiosity decreases at a rate that is also incapable of triggering PB, then even high benefits of CI appear insufficient to produce thriving societies. This lends plausibility to the theory of prosocial equilibrium [23], and complements other empirical findings related to religion and anxiety [57,58].
- If PB is easily triggered (0.001 on x-facet), societies decline in religiosity only if they easily forget about religiosity (>=0.151 on x-axis), threats are low to medium (six top rows on y-facet), and PP is high (0.2–0.35, circle/triangle shapes). These results also point out the importance of prosocial behaviour for keeping high levels of religiosity (via reinforcing religious beliefs). If PB is easily triggered, it will not only help societies thrive but will also maintain religiosity even in the face of medium or high threats. It is only when parochial prosociality becomes high and forgetting is high that religiosity declines. This highlights the resilience of religiosity across a wide variety of scenarios.
- When threat is medium-high (0.05 on y-facet), religiosity decreases if the CI benefit is high (0.35–0.45, blue/purple colours), PP is medium-high (circle/triangle/square shapes), PB is not so easily triggered (> 0.001 on x-facet), and religiosity is somewhat easy to forget (x-axis). This result underscores the relevance of effective CI in the decline of religiosity, under medium-high threat scenarios. If CI do not benefit the society enough, then religiosity will not decline. This finding complements other research that has shed light on the ways in which (and extent to which) well-functioning secular institutions play a role in the decline of religiosity [59–62].

## Discussion

In this study, we provided a theoretical exposition of the prosocial equilibrium and existential security theories. Our findings demonstrated that the interplay between anxiety, prosociality, and religion, as outlined in the prosocial equilibrium theory, can lead to the emergence of successful religious societies, but this occurs within a specific range of parameter values and under particular conditions. Furthermore, our findings reveal that while the timing of reproduction influences the number of successful religious societies that emerge, the underlying conditions for their development remain consistent regardless of the reproduction schedule. Additionally, when central institutions are introduced, they can lead to the formation of secular societies. However, the pace of secularisation varies widely depending on specific conditions, and some conditions allow for the coexistence of central institutions and religiosity, with certain scenarios even resulting in increased religiosity. Overall, our agent-based simulation study underscores the plausibility of both theories and offers valuable insights into the potential explanations and underlying conditions that contribute to the formation of thriving religious and non-religious societies.

Our model builds on the ideas of Malinowski [14,15] and Durkheim [1], synthesized in the prosocial equilibrium theory [23]. Malinowski argued that rituals are primarily caused by anxiety felt by individuals. In contrast, Durkheim emphasized the role of religiosity in promoting social integration, moral regulation, and the maintenance of social order by fostering unity and solidarity among society members. The prosocial equilibrium theory bridges these perspectives by proposing a feedback loop between anxiety, religiosity, and prosocial behavior [23]. Our model not only demonstrates the plausibility of this dynamic but also reveals the necessary conditions for the emergence of thriving religious societies.

Once we managed to create thriving religious societies in our model, we started exploring the role of central institutions on the secularisation process. As argued in existential security theory [34], in our model, central institutions function as a welfare state by alleviating the insecurity experienced by individuals due to threats (i.e., analogous to providing basic needs, social and economic support, protection, etc.). Our aim was to examine the conditions under which central institutions could lead to secularisation. In this light, two key parameters take on added significance. Firstly, it becomes clear that even where secular institutions lead to a pattern of secularisation, this pattern can be potentially overwhelmed by sufficiently large threats that those institutions will be insufficient to deal with. This was vividly exemplified during the COVID-19 crisis, which saw notable spikes in religiosity early in the pandemic. Religious beliefs played a stronger role in people’s lives and religious behaviours such as attendance and prayer increased in response to anxiety about the pandemic [21,63]. Secondly, much depends upon how readily people will engage in religious motivated (parochial) prosocial behaviour – so we can expect religious traditions that are more successful in motivating such behaviour, such as those invoking moralizing supernatural agents [64], remaining more relevant in societies with strong secular institutions.

Beyond these two key parameters, we have observed a complex set of relations among the variables in the model and factors influencing whether thriving societies with declining religiosity are likely to form. In particular, the model helps to explain the mechanism behind the rapid loss of religiosity, such as the effectiveness of secular institutions in maintaining security [34], and the lack of exposure to religiously motivated prosocial behaviour [26,28], and the highly parochial nature of prosocial behaviour between religious individuals of the same group [65,66]. What is interesting to note in general is how specific, across the range of the possible values in the model, the combinations of values need to be for successful irreligious societies to form. Of course, most secularised societies have birth rates below replacement level [67] and therefore, would not be classified as thriving in this model, so the difficulty for such societies to be formed in the model fits with real world evidence. Having said this, the study appears to bear out its key theoretical starting point, i.e., that by ensuring high security levels, secularised societies may serve to undermine a previously existing prosocial equilibrium where high levels of religiosity were maintained.

Given the limited scope of our model, it’s important to note that several theories related to the emergence, acquisition, and diffusion of religious beliefs were not explicitly addressed but abstracted in our model’s processes. For instance, established research indicates that religions featuring moralizing and punitive gods tend to more effectively motivate prosocial behaviours compared to religions with other types of deities [64]. Moreover, intergroup competition often plays a significant role in the propagation of these religious beliefs, as societies adhering to such beliefs may either conquer other groups or influence their assimilation [68,69]. In our model, we opted not to delve into these specific processes, as our primary focus was not on intergroup competition or the competition among religions with differing types of gods. Instead, we concentrated on examining the dynamics of religiosity, anxiety, and prosocial behaviour. Consequently, we assumed that religiosity in our model aligns with the kind associated with moralizing, punishing gods; and we abstracted intergroup competition through the threats experienced by individuals.

Similarly, Credibility Enhancing Display (CRED) theory [26] is a well-supported theory explaining the spread of religious beliefs. In our model, this mechanism was abstracted within the process whereby prosocial behaviour increases religiosity among both performers and receivers, with prosocial behaviour being an example of a CRED. Likewise, Fuzzy Fidelity Theory emphasizes the intergenerational nature of the secularisation process and underscores the significance of formative years, particularly the ages between 12 and 25, in shaping religious beliefs [30]. Although not explicitly modelled, our framework takes these assumptions into account. For instance, the process where individuals experience a decline in religiosity over time is influenced by the idea that this process stabilizes once individuals reach 25 years of age. Our main objective here was not to comprehensively model all the factors and processes indicated in these theories and their interactions, but rather, we primarily concentrated on exploring the Prosocial Equilibrium Theory. Nonetheless, these aspects offer natural model extensions that can be explored in future studies.

Further, many other aspects relevant to the relationship between religiosity and security were either not explored or largely abstracted in the model. While a large number of potential causal connections could be postulated between the modelled variables, it was important to limit the simulation to a small number deemed most important at the long-time scales in question. Religiosity and insecurity could be connected, but via what mechanisms? Similarly, religiosity and anxiety could also be connected, but how? In the model, religiosity is understood as a one-dimensional abstraction indicative of how religious someone is, though in reality, religiosity consists of many interconnected behavioural and belief traits. We have singled out religiously motivated prosocial behaviour as a distinct trait because of its critical role in our study. It ties both anxiety and general religiosity, and also impacts insecurity. In numerous theories, ritual behaviour is similarly emphasised, and is often very closely connected to prosocial behaviour, but rituals lack the direct impact on the community’s material conditions that explicitly prosocial behaviour has [20–22]. In other words, we think that religiosity is affected by anxiety (via engaging in/witnessing prosocial behaviour in our model but via engaging in/witnessing rituals more generally). Put it yet another way, anxiety changes behavioural aspects of overall religiosity, thereby leading to long-term changes in overall religiosity. Correlational studies may detect connections among these variables [27–29] but will not necessarily reveal the underlying mechanisms involved. A model thus provides much more insight into the issue by showing the consequences of specific assumptions.

As a result of making these concrete design decisions during the process of creating the agent-based model we feel that we have been able to arrive at clear conclusions about the underlying theories. Firstly, in the initial stage of the model without the introduction of CI, we were able to show that something like a prosocial equilibrium (per Talmont-Kaminski) can be maintained under conditions that are qualitatively plausible. This allowed us to solve the seeming contradiction between Malinowski’s claim that anxiety leads to engaging in religious behaviour [15] and Durkheim’s view that religions promote cooperation that over time will lead to security and lower anxiety [1] – a fortuitous result given the broad empirical support both these theories have. In addition, by introducing into our model central institutions that enforced cooperation, we saw how this equilibrium could be undermined by mechanisms that maintained security in a society independently of the religiosity of the population, thereby providing a clear mechanism for existential security theory [34]. This result, together with the patterns of interactions between parameters set out in Fig 8, suggests concrete lines of empirical investigation into these phenomena.

However, as already noted, while the relationships between the variables have been established, determining the exact positioning of real societies within the vast possibility space is challenging. For instance, it remains unverified how the model-suggested values for triggering prosocial behaviours and increasing anxiety, religiosity, threats, and so forth align with real-world counterparts. This is particularly significant given that in many sets of conditions, central institutions were not sufficient to lead to the kind of secularisation that many western societies have witnessed. Also, social structure was treated in very simplistic ways. It lacked features such as kin-based structures (e.g., clans or chiefdoms), which meant that small, intensely cooperative subgroups could not form. Finally, the reference model we used assumed slow population growth, whereas it is far from clear that such societies would not either die out or experience explosive growth. These simplifications were necessary as an initial step to elucidate the interplay between variables. As a result, our simulation experiments have been able to generate an unambiguous bottom-up explanation of conditions that give rise to highly religious societies and the subsequent decline in religiosity in the context of our model. Now that the model has been verified and validated, future work might extend the architecture to include other theories, building on the insights provided here. Such extensions would help elucidate other fundamental mechanisms such as competing or complementary explanations.

## Supporting information

S1 Text Additional simulation results. (DOCX)

S2 Text Full ODD + D protocol of the agent-based model. (DOCX)
