# Supplementary material for: The rise and fall of religion: An agent-based model of secularisation, security and prosociality
Source: PLoS One. 2025 Nov 20;20(11):e0327674. doi: 10.1371/journal.pone.0327674 (PMC12633920; doi:10.1371/journal.pone.0327674)
Supplement: S1 Text — (DOCX) [file pone.0327674.s001.docx]

# Supporting information of the MS: “The Rise and Fall of Religion: An Agent-Based Model of Secularisation, Security and Prosociality”

## The effect of stochastic threats

To explore whether stochastic threats had some effect on the models result, we modified the way threats happened in the model while keeping the same parameter space of the 3^rd^ analysis (Table 2 in main MS). We changed the constant yearly increase of threat to (1) a given number of threats per year determined from a Poisson distribution (lambda varied between 0 and 10) and (2) the intensity value of each threat draw from an exponential distribution function (with lambda fixed at 10) (Figure 1). After running the simulations with the stochastic threats implementation, results remained qualitatively the same: percentage of societies with pop sizes > 2500: 31.80%, 79.80%, and 15.72% when the logical order of reproduction was random, before, after prosocial behaviour (PB) respectively.

| A) | B) |
| --- | --- |
| 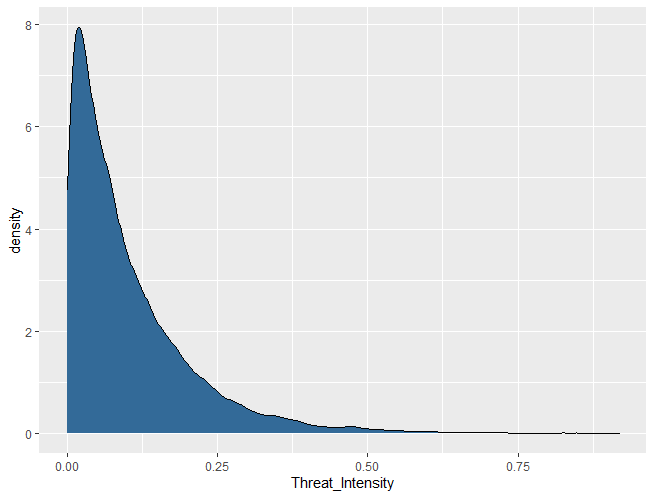 | 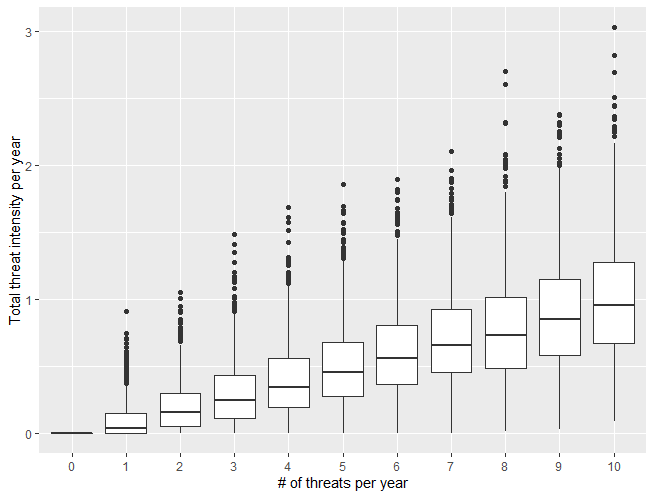 |

Figure 1 Stochastic threats. A) Density plot for threat intensity (x-axis), most threats are of low intensity, i.e., <0.25. B) Boxplots of the total threat experienced per year according to different values of lambda (mean number of threats per year)

## The effect of parochial prosociality (PP)

We implemented parochial prosociality (PP) in the model and studied its effects on population grow and levels of religiosity. When PP is on, agents performing a PB benefit neighbors only if those neighbors have a religiosity equal or higher to the performing agent’s religiosity - PP value. Thus, the lower the values of PP, the higher the similarity in religiosity needed among agents to receive the benefit of PB. Table 1 shows the parameter space used when exploring the effect of PP.

|  | MIN | MAX |
| --- | --- | --- |
| PB threshold | 0.001 | 0.050 |
| PB inc rel self | 0.100 | 0.500 |
| PB inc rel neigh | 0.100 | 0.500 |
| PB dec insec self | 0.250 | 0.500 |
| PB dec insec neigh | 0.250 | 0.500 |
| PB wellbeing cost | 0.001 | 0.025 |
| Num Neigh Benefited | 5.000 | 10.000 |
| Threat value | 0.001 | 0.500 |
| Rel Dec Perc | 0.001 | 0.500 |
| Parochial Prosociality | 0.010 | 1.000 |

Table 1 Parameter Space when studying effect of Parochial Prosociality

Results show that PP reduces the % of growing societies, i.e., societies with > 2500 agents at year 600 (Table 2). This is expected because when PP is on, the benefit of PB is received by a lower number of agents and thus the growth of societies is reduced. Indeed, at low values of PP, the percentage of successful societies is lower than expected from the distribution of PP values in the parameter sampling, but as the value of PP increases the percentage of successful societies becomes higher than expected from the parameter sampling (Table 3).

| Reproduction occurring | Non-parochial | Parochial Prosociality |
| --- | --- | --- |
| After | 24.12% | 17.92% |
| Random | 44.96% | 32.46% |
| Before | 74.14% | 53.06% |

Table 2 Percentage of societies with populations > 2500 agents

| PP value range | After | | | Random | | | Before | | |
| --- | --- | --- | --- | --- | --- | --- | --- | --- | --- |
|  | % Obs | % Exp | Diff | % Obs | % Exp | Diff | % Obs | % Exp | Diff |
| [0.0-0.1] | 3.27% | 10.00% | -6.73% | 2.58% | 10.00% | -7.42% | 0.65% | 10.00% | -9.35% |
| [0.1-0.2] | 3.64% | 10.00% | -6.36% | 4.39% | 10.00% | -5.61% | 1.47% | 10.00% | -8.53% |
| [0.2-0.3] | 7.64% | 10.00% | -2.36% | 6.21% | 10.00% | -3.79% | 3.54% | 10.00% | -6.46% |
| [0.3-0.4] | 7.27% | 10.00% | -2.73% | 8.18% | 10.00% | -1.82% | 6.02% | 10.00% | -3.98% |
| [0.4-0.5] | 10.18% | 10.00% | 0.18% | 11.82% | 10.00% | 1.82% | 11.50% | 10.00% | 1.50% |
| [0.5-0.6] | 11.27% | 10.00% | 1.27% | 11.06% | 10.00% | 1.06% | 13.57% | 10.00% | 3.57% |
| [0.6-0.7] | 15.27% | 10.00% | 5.27% | 13.18% | 10.00% | 3.18% | 15.93% | 10.00% | 5.93% |
| [0.7-0.8] | 18.55% | 10.00% | 8.55% | 14.09% | 10.00% | 4.09% | 15.46% | 10.00% | 5.46% |
| [0.8-0.9] | 13.09% | 10.00% | 3.09% | 13.79% | 10.00% | 3.79% | 15.63% | 10.00% | 5.63% |
| [0.9-1.0] | 9.82% | 10.00% | -0.18% | 14.70% | 10.00% | 4.70% | 16.22% | 10.00% | 6.22% |

Table 3 Percentage of successful societies Observed and Expected within an specific parameter range of PP

Further, in those societies with PP on and populations above 2500 agents at year 600, the level of average religiosity is much higher than when PP is off (Table 4). It seems, thus, that PP reduces the proportion of successful societies to those with high religiosity.

|  | Non-parochial | | | Parochial Prosociality | | |
| --- | --- | --- | --- | --- | --- | --- |
|  | Rel > 0.5 | Rel > 0.75 | Rel > 0.9 | Rel > 0.5 | Rel > 0.75 | Rel > 0.9 |
| After | 96.93% | 84.49% | 42.53% | 100.00% | 94.86% | 62.16% |
| Random | 97.50% | 87.36% | 49.28% | 100.00% | 95.68% | 63.03% |
| Before | 98.40% | 85.02% | 39.68% | 99.92% | 87.71% | 40.89% |

Table 4 Successful societies (pop size > 2500) and average religiosity


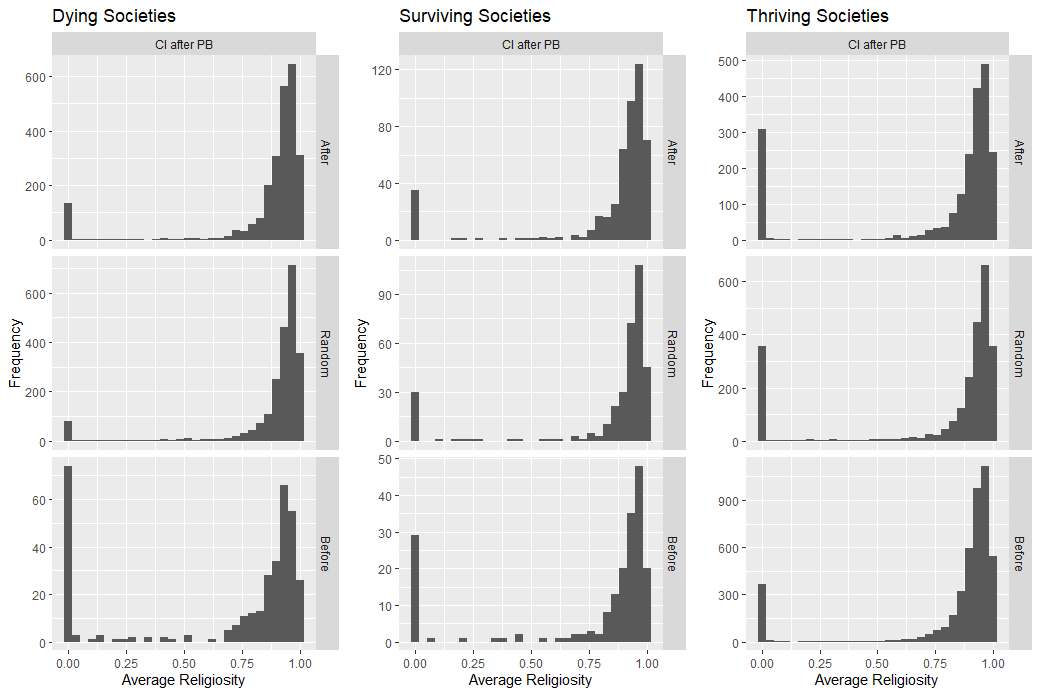


Figure 2. Average Religiosity at year 600.

Note that scales in the y-axes are different among the different reproduction times. This presentation highlights that the pattern is similar among the conditions despite different absolute values.


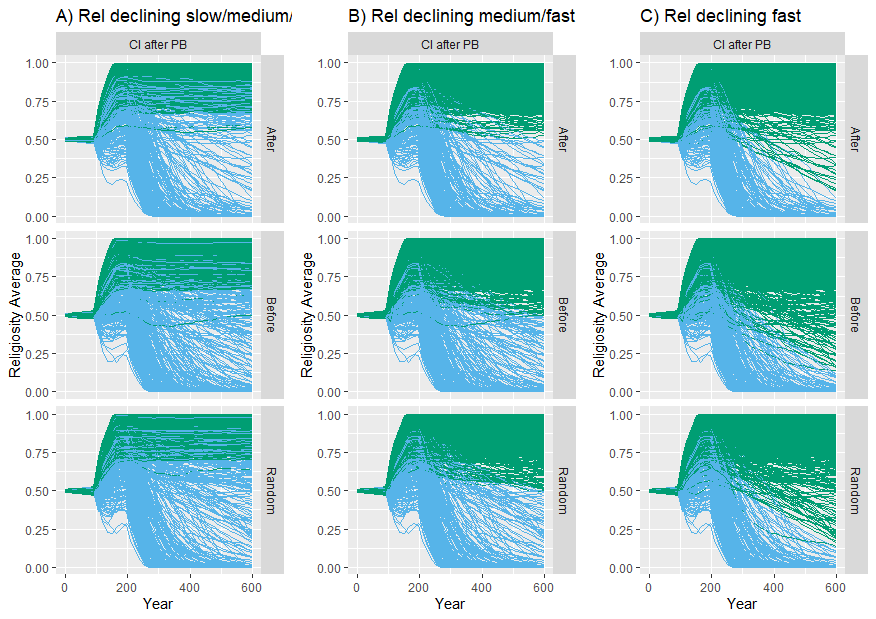


Figure 3. Decline of religiosity at different paces. Blue lines are societies where religiosity was declining during period 200-600 years and were the target of the sensitivity assessor. A) Religiosity declining at a slow/medium/fast pace; B) Religiosity declining at a medium/fast pace; C) Religiosity declining at a fast pace.

|  | **Declining Rel S/M/F** | | | **Declining Rel M/F** | | | **Declining Rel F** | | |
| --- | --- | --- | --- | --- | --- | --- | --- | --- | --- |
| **CONDITIONS** | **After** | **Random** | **Before** | **After** | **Random** | **Before** | **After** | **Random** | **Before** |
| **1) Yearly threat AND PB threshold** |  |  |  |  |  |  |  |  |  |
| Lower half AND Lower half | -6% | -6% | 3% | -8% | -9% | 3% | -8% | -9% | 3% |
| Lower half AND Upper half | 27% | 26% | 36% | 33% | 33% | 45% | 32% | 34% | 48% |
| Upper half AND Lower half | -10% | -10% | -21% | -12% | -13% | -25% | -12% | -13% | -25% |
| Upper half AND Upper half | -11% | -10% | -18% | -13% | -11% | -23% | -13% | -12% | -25% |
| **2) Yearly threat AND PB WB cost** |  |  |  |  |  |  |  |  |  |
| Lower half AND Lower half | 5% | 8% | 18% | 4% | 6% | 20% | 3% | 6% | 20% |
| Lower half AND Upper half | 16% | 12% | 21% | 21% | 17% | 28% | 22% | 18% | 30% |
| Upper half AND Lower half | -15% | -14% | -21% | -17% | -16% | -26% | -17% | -17% | -27% |
| Upper half AND Upper half | -6% | -6% | -18% | -7% | -8% | -23% | -7% | -8% | -24% |
| **3) Yearly threat AND Rel Dec Perc** |  |  |  |  |  |  |  |  |  |
| Lower half AND Lower half | 9% | 4% | 14% | 13% | 6% | 20% | 14% | 11% | 24% |
| Lower half AND Upper half | 12% | 16% | 25% | 12% | 17% | 28% | 10% | 14% | 27% |
| Upper half AND Lower half | -12% | -12% | -25% | -12% | -13% | -25% | -12% | -13% | -25% |
| Upper half AND Upper half | -9% | -8% | -14% | -12% | -11% | -24% | -12% | -12% | -26% |
| **4) Yearly threat AND Parochial Prosociality** |  |  |  |  |  |  |  |  |  |
| Lower half AND Lower half | 17% | 18% | 27% | 14% | 14% | 28% | 13% | 13% | 25% |
| Lower half AND Upper half | 5% | 2% | 12% | 10% | 9% | 21% | 11% | 12% | 25% |
| Upper half AND Lower half | -9% | -7% | -14% | -12% | -11% | -23% | -12% | -12% | -25% |
| Upper half AND Upper half | -12% | -13% | -25% | -12% | -13% | -25% | -12% | -13% | -25% |
| **5) Yearly threat AND CI WB cost** |  |  |  |  |  |  |  |  |  |
| Lower half AND Lower half | 11% | 11% | 19% | 13% | 10% | 25% | 11% | 11% | 26% |
| Lower half AND Upper half | 10% | 10% | 20% | 11% | 13% | 23% | 13% | 14% | 24% |
| Upper half AND Lower half | -17% | -15% | -20% | -19% | -19% | -26% | -19% | -19% | -27% |
| Upper half AND Upper half | -4% | -5% | -19% | -5% | -5% | -23% | -5% | -5% | -23% |
| **6) Yearly threat AND CI benefit** |  |  |  |  |  |  |  |  |  |
| Lower half AND Lower half | 0% | 1% | 12% | -7% | -4% | 7% | -8% | -4% | 7% |
| Lower half AND Upper half | 21% | 20% | 27% | 31% | 28% | 42% | 32% | 29% | 44% |
| Upper half AND Lower half | -7% | -5% | -21% | -7% | -6% | -23% | -7% | -6% | -23% |
| Upper half AND Upper half | -14% | -15% | -18% | -17% | -18% | -26% | -17% | -19% | -28% |
| **7) PB threshold AND PB WB cost** |  |  |  |  |  |  |  |  |  |
| Lower half AND Lower half | -13% | -11% | -11% | -18% | -17% | -15% | -19% | -18% | -16% |
| Lower half AND Upper half | -3% | -5% | -7% | -2% | -4% | -7% | -1% | -4% | -6% |
| Upper half AND Lower half | 3% | 5% | 8% | 5% | 8% | 10% | 5% | 8% | 9% |
| Upper half AND Upper half | 13% | 11% | 10% | 15% | 14% | 12% | 15% | 14% | 13% |
| **8) PB threshold AND Rel Dec Perc** |  |  |  |  |  |  |  |  |  |
| Lower half AND Lower half | -11% | -13% | -15% | -10% | -13% | -12% | -10% | -11% | -11% |
| Lower half AND Upper half | -5% | -3% | -3% | -10% | -9% | -10% | -10% | -11% | -12% |
| Upper half AND Lower half | 8% | 5% | 4% | 11% | 7% | 8% | 12% | 9% | 9% |
| Upper half AND Upper half | 8% | 11% | 14% | 9% | 15% | 14% | 8% | 13% | 13% |
| **9) PB threshold AND Parochial Prosociality** |  |  |  |  |  |  |  |  |  |
| Lower half AND Lower half | -1% | 0% | -2% | -6% | -7% | -8% | -7% | -9% | -10% |
| Lower half AND Upper half | -15% | -16% | -16% | -14% | -15% | -14% | -13% | -13% | -12% |
| Upper half AND Lower half | 9% | 11% | 15% | 8% | 11% | 12% | 8% | 10% | 9% |
| Upper half AND Upper half | 7% | 5% | 3% | 12% | 11% | 10% | 12% | 12% | 13% |
| **10) PB threshold AND CI WB cost** |  |  |  |  |  |  |  |  |  |
| Lower half AND Lower half | -13% | -11% | -9% | -15% | -15% | -11% | -15% | -15% | -12% |
| Lower half AND Upper half | -4% | -5% | -9% | -5% | -7% | -11% | -4% | -7% | -11% |
| Upper half AND Lower half | 7% | 6% | 9% | 8% | 6% | 11% | 7% | 7% | 11% |
| Upper half AND Upper half | 9% | 10% | 9% | 12% | 15% | 11% | 13% | 16% | 11% |
| **11) PB threshold AND CI benefit** |  |  |  |  |  |  |  |  |  |
| Lower half AND Lower half | -9% | -8% | -11% | -10% | -9% | -12% | -11% | -10% | -13% |
| Lower half AND Upper half | -7% | -8% | -7% | -10% | -12% | -10% | -9% | -12% | -9% |
| Upper half AND Lower half | 2% | 4% | 2% | -4% | 0% | -4% | -5% | 0% | -3% |
| Upper half AND Upper half | 14% | 13% | 16% | 24% | 22% | 26% | 25% | 23% | 25% |
| **12) PB WB cost AND Rel Dec Perc** |  |  |  |  |  |  |  |  |  |
| Lower half AND Lower half | -7% | -8% | -8% | -8% | -10% | -7% | -9% | -9% | -7% |
| Lower half AND Upper half | -2% | 2% | 6% | -5% | 0% | 1% | -6% | -1% | 0% |
| Upper half AND Lower half | 5% | 0% | -3% | 9% | 4% | 2% | 10% | 7% | 5% |
| Upper half AND Upper half | 5% | 6% | 5% | 4% | 6% | 3% | 4% | 3% | 2% |
| **13) PB WB cost AND Parochial Prosociality** |  |  |  |  |  |  |  |  |  |
| Lower half AND Lower half | 1% | 3% | 5% | -5% | -4% | -1% | -7% | -7% | -5% |
| Lower half AND Upper half | -10% | -9% | -8% | -8% | -5% | -4% | -7% | -4% | -2% |
| Upper half AND Lower half | 7% | 9% | 8% | 8% | 8% | 6% | 8% | 8% | 5% |
| Upper half AND Upper half | 3% | -3% | -6% | 5% | 2% | 0% | 6% | 3% | 2% |
| **14) PB WB cost AND CI WB cost** |  |  |  |  |  |  |  |  |  |
| Lower half AND Lower half | -8% | -5% | 0% | -9% | -8% | -1% | -11% | -9% | -2% |
| Lower half AND Upper half | -2% | -1% | -2% | -4% | -1% | -4% | -3% | -2% | -5% |
| Upper half AND Lower half | 2% | 1% | -1% | 2% | 0% | 1% | 3% | 0% | 2% |
| Upper half AND Upper half | 7% | 5% | 3% | 11% | 10% | 5% | 12% | 10% | 5% |
| **15) PB WB cost AND CI benefit** |  |  |  |  |  |  |  |  |  |
| Lower half AND Lower half | -8% | -6% | -5% | -10% | -7% | -8% | -11% | -8% | -9% |
| Lower half AND Upper half | -2% | 0% | 3% | -3% | -2% | 3% | -3% | -3% | 2% |
| Upper half AND Lower half | 0% | 1% | -4% | -5% | -3% | -8% | -5% | -3% | -7% |
| Upper half AND Upper half | 9% | 5% | 6% | 18% | 12% | 13% | 19% | 13% | 14% |
| **16) Rel Dec Perc AND Parochial Prosociality** | | | | | | | |  |  |
| Lower half AND Lower half | -1% | -3% | -5% | 2% | -2% | -1% | 1% | -1% | 0% |
| Lower half AND Upper half | -1% | -6% | -6% | -1% | -4% | -4% | 1% | -1% | -2% |
| Upper half AND Lower half | 9% | 14% | 19% | 1% | 6% | 5% | 0% | 2% | -1% |
| Upper half AND Upper half | -6% | -6% | -8% | -1% | 0% | 0% | -2% | 0% | 2% |
| **17) Rel Dec Perc AND CI WB cost** |  |  |  |  |  |  |  |  |  |
| Lower half AND Lower half | -6% | -7% | -6% | -3% | -6% | -2% | -3% | -4% | 0% |
| Lower half AND Upper half | 3% | -1% | -5% | 4% | 0% | -3% | 5% | 2% | -1% |
| Upper half AND Lower half | 0% | 2% | 6% | -4% | -3% | 1% | -5% | -5% | -1% |
| Upper half AND Upper half | 3% | 6% | 5% | 3% | 9% | 3% | 4% | 7% | 2% |
| **18) Rel Dec Perc AND CI benefit** |  |  |  |  |  |  |  |  |  |
| Lower half AND Lower half | -4% | -5% | -7% | -6% | -7% | -9% | -6% | -6% | -8% |
| Lower half AND Upper half | 1% | -3% | -4% | 7% | 1% | 5% | 8% | 4% | 7% |
| Upper half AND Lower half | -3% | 0% | -2% | -8% | -2% | -7% | -9% | -4% | -8% |
| Upper half AND Upper half | 6% | 8% | 13% | 8% | 8% | 11% | 8% | 6% | 9% |
| **19) Parochial Prosociality AND CI WB cost** |  |  |  |  |  |  |  |  |  |
| Lower half AND Lower half | 4% | 6% | 8% | -3% | -2% | 2% | -4% | -4% | -1% |
| Lower half AND Upper half | 4% | 5% | 6% | 5% | 6% | 2% | 6% | 5% | 1% |
| Upper half AND Lower half | -10% | -11% | -8% | -4% | -6% | -2% | -4% | -5% | 1% |
| Upper half AND Upper half | 2% | 0% | -5% | 2% | 3% | -2% | 3% | 4% | 0% |
| **20) Parochial Prosociality AND CI benefit** |  |  |  |  |  |  |  |  |  |
| Lower half AND Lower half | -2% | 1% | -1% | -7% | -5% | -8% | -8% | -6% | -9% |
| Lower half AND Upper half | 9% | 10% | 15% | 10% | 9% | 12% | 9% | 7% | 9% |
| Upper half AND Lower half | -5% | -6% | -8% | -7% | -5% | -8% | -8% | -4% | -6% |
| Upper half AND Upper half | -2% | -6% | -5% | 5% | 1% | 4% | 7% | 3% | 7% |
| **21) CI WB cost AND CI benefit** |  |  |  |  |  |  |  |  |  |
| Lower half AND Lower half | -5% | -3% | -6% | -9% | -6% | -8% | -10% | -8% | -7% |
| Lower half AND Upper half | 0% | -1% | 5% | 2% | -2% | 7% | 2% | -1% | 7% |
| Upper half AND Lower half | -2% | -1% | -4% | -5% | -3% | -8% | -5% | -3% | -8% |
| Upper half AND Upper half | 8% | 6% | 4% | 12% | 12% | 8% | 13% | 11% | 9% |

Table 5. Percentage difference (Observed – Expected) of societies with declining religiosity at different paces and within a specific combination of parameters range. CI= Central Institution, WB= Wellbeing, PB= Prosocial Behaviour. Color scale goes from dark yellow (negative) to dark green (positive). Positive (dark green) values mean that more TSDR were present than expected and vice versa for negative (dark yellow) values. Note that the color scale is adjusted to the percentage range within each combination of parameters.

| **Varied parameters** | **Set of values** | **# values** |
| --- | --- | --- |
| PB threshold | [0.001, 0.011, 0.021, 0.031, 0.041, 0.051] | 6 |
| Threat value | [0.001, 0.002, 0.003, 0.004, 0.005, 0.006, 0.010, 0.050, 0.100, 0.150] | 10 |
| Rel Dec Perc | [0.001, 0.051, 0.101, 0.151, 0.201, 0.251] | 6 |
| Parochial Prosociality | [0.200, 0.350, 0.500, 0.650, 0.800, 0.950] | 6 |
| CI Benefit | [0.050, 0.150, 0.250, 0.350, 0.450] | 5 |
| **Fixed parameters** | **Value** |  |
| PB inc rel self | 0.275 | 1 |
| PB inc rel neigh | 0.275 | 1 |
| PB dec insec self | 0.375 | 1 |
| PB dec insec neigh | 0.375 | 1 |
| PB wellbeing cost | 0.015 | 1 |
| Num Neigh Benefited | 7 | 1 |
| CI WB cost | 0.025 | 1 |

Table 6 Parameter space for final exploration of conditions leading to declining religiosity in thriving societies.
This table presents the specific parameter combinations used to systematically investigate how variations in five key parameters—PB threshold, threat intensity, yearly decrease in religiosity, parochial prosociality, and CI benefit—affect religiosity trajectories. All other parameters were held constant to isolate the effects of these variables. Results are reported in Tables 9 and 10 and visualized in Figure 8 of the main manuscript.


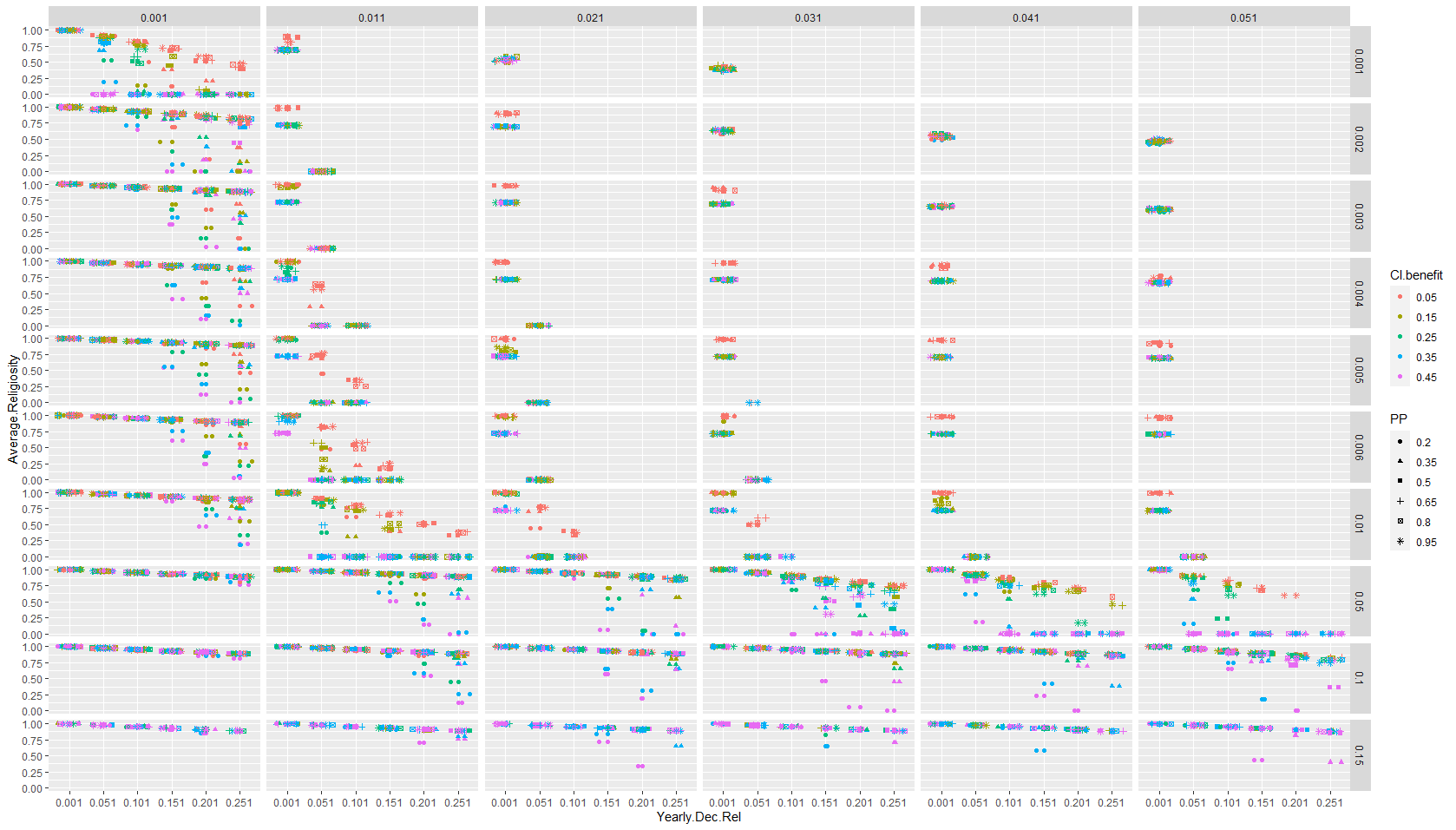


Figure 4. Average religiosity of thriving societies according to different parameters’ values and when reproduction occurs at random.

Data points are jittered along the x-axis for best visualization


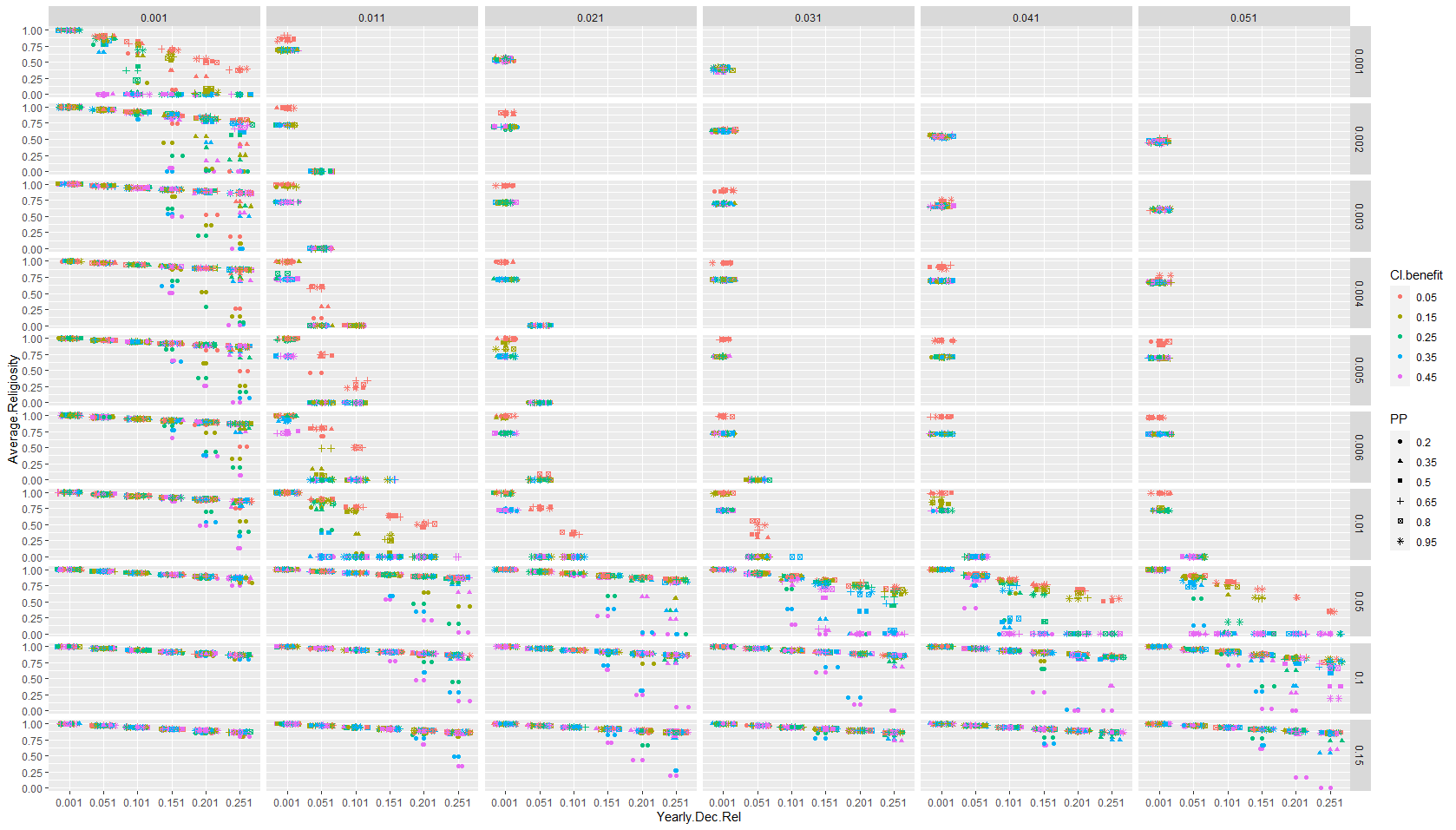


Figure 5. Average Religiosity of thriving societies according to different parameters’ values and when reproduction occurs before PB.

Data points are jittered along the x-axis for best visualization
