# Supplementary material for: The rise and fall of religion: An agent-based model of secularisation, security and prosociality
Source: PLoS One. 2025 Nov 20;20(11):e0327674. doi: 10.1371/journal.pone.0327674 (PMC12633920; doi:10.1371/journal.pone.0327674)
Supplement: S2 Text — (DOCX) [file pone.0327674.s002.docx]

# ODD+D protocol of the Prosociality Model of Religion.

| 1. Overview | I.i  Purpose | | I.i.a What is the purpose of the study? | To explore the relationship between anxiety, environmental threats, and religiosity at the individual and societal level. |
| --- | --- | --- | --- | --- |
|  |  |  | I.i.b For whom is the model designed? | For social and cognitive scientists of religion. |
|  | I.ii  Entities, state varia-bles and scales | | I.ii.a What kinds of entities are in the model? | Human agents |
|  |  |  | I.ii.b By what attributes (i.e. state variables and parameters) are these entities characterized? | Humans:   1. Age 2. Gender 3. Marital status 4. Religiosity 5. Wellbeing 6. Insecurity 7. Sensitivity 8. Anxiety   Global parameters  Initialization:   1. Initial population (n=1000) 2. Initial insecurity Mean population (0.5) 3. Initial insecurity Standard deviation (0.1) 4. Initial religiosity Mean population (0.5) 5. Initial religiosity Standard deviation (0.1) 6. Initial sensitivity Mean population (0.5) 7. Initial sensitivity Standard deviation (0.1)   Reproduction (see submodels)   1. Reproduction cost on wellbeing 2. Reproduction threshold 3. Probability curve shape 4. Importance of Insecurity 5. Importance of wellbeing   Wellbeing and age dynamics (see submodels)   1. Age threshold for wellbeing decline 2. Equation intercept 3. Exponent of the increase-wellbeing equation 4. Exponent of the decrease-wellbeing equation   Wellbeing and insecurity dynamics (see submodels)   1. Threshold of decrease 2. Maximum increase in wellbeing 3. Maximum decrease in wellbeing   Prosocial behavior (see submodels)   1. Threshold for triggering prosocial behavior 2. Self-increase in security after performing prosocial behavior 3. Neighbor increase in security after receiving prosocial behavior 4. Self-increase in religiosity after performing prosocial behavior 5. Neighbor increase in religiosity after receiving prosocial behavior 6. Number of neighbors benefited with prosocial behavior 7. Prosocial behavior cost on wellbeing   Threats   1. Rate of threats per year (Poisson distribution) 2. Intensity of threats per year (Exponential distribution)   Other   1. Max age difference for Marriage 2. Yearly religiosity decrease |
|  |  |  | I.ii.c What are the exogenous factors / drivers of the model? | ***Environmental threats:*** Every year, agents experience a number of threats of different intensity. The number and intensity of threats increase the insecurity of agents. The increase of insecurity may have a cascade of effects on wellbeing, reproduction, religiosity, and prosocial behavior. |
|  |  |  | I.ii.d If applicable, how is space included in the model? | A grid of 500x500 of continuous coordinates. At initialization agents are located at random. |
|  |  |  | I.ii.e What are the temporal and spatial resolutions and extents of the model? | Yearly time steps; simulations are run for 500 years. |
|  | I.iii  Process overview and scheduling | | I.iii.a What entity does what, and in what order? | ***Annual events***  Every year, agents experience a number of threats of different intensity. The number and intensity of threats increase the insecurity of agents. The increase of insecurity may have a cascade of effects on wellbeing, reproduction, religiosity, and prosocial behavior.   1. Agents age by one year 2. Agents increase/reduce their *wellbeing* according to their age 3. Threats and threats’ intensity are generated and experienced. 4. Agents increase their *insecurity* according to the number and intensity of threats 5. Agents try to reproduce if they are female, married, and in age reproduction (note that reproduction can also happen (a) after 6 and 7 or (b) at random (50% chance before or after 6 and 7 respectively) 6. Agents behave prosocially if the *threshold of prosocial behavior* is exceeded 7. Agents increase/reduce their *wellbeing* according to their value of insecurity 8. Agents search for a potential partner if they are ≥ 15 y.o. and not married 9. Agents 25 y.o and younger decrease their *religiosity* according to *Yearly religiosity decrease* 10. Agents may die depending on their *wellbeing* level |
| 1. Design Concepts | II.i  Theoretical and Empirical Background | | II.i.a Which general concepts, theories or hypotheses are underlying the model’s design at the system level or at the level(s) of the submodel(s) (apart from the decision model)? What is the link to complexity and the purpose of the model? | **Prosocial equilibrium theory**:  The connection between religion and cooperation as well as the connection between anxiety and religion potentially form two parts of a negative feedback mechanism that could underpin a prosocial equilibrium, the connection between cooperation and anxiety forming the final element (Talmont-Kamisnky 2014). The picture is that of environmental threats leading to increased anxiety and thereby to increased religiosity. However, an increase in religiosity drives increased cooperation, which helps the society deal with the threats and thereby lower anxiety. In effect, a relatively high level of religiosity and cooperation is maintained, allowing large-scale societies to thrive. |
|  |  |  | II.i.b On what assumptions is/are the agents’ decision model(s) based? | Prosocial behavior is trigger when the product of anxiety times religion is above the *threshold of prosocial behavior*. The decision to reproduce depends on the wellbeing and insecurity values of the partners. The decision of decreasing religiosity yearly up to 25 years old is based on empirical data. In survey data, it is observed that after 25 years old, the religiosity level of individuals neither increases nor decreases. The minimum age for marriage and age range of reproduction are informed by sociodemographic data from the UN World Population Prospects (https://population.un.org/wpp/Download/Standard/Population/)  The neighbors benefited from prosocial behaviors are selected according to proximity to the agent. This is based in common sense. |
|  |  |  | II.i.c Why is /are certain decision model(s) chosen? | After a prosocial behavior agents increase their security and religiosity.  After a prosocial behavior agents increase the security and religiosity of their neighbors.  The neighbors benefited by a prosocial behavior are selected based on proximity to the agent performing the prosocial behavior. This is based on common sense.  The decision for a minimum age of marriage and the age of reproduction range follow the ages in the World Population Prospects of the UN. |
|  |  |  | II.i.d If the model / submodel (e.g. the decision model) is based on empirical data, where do the data come from? | The data comes from the World Population Prospects of the UN (https://population.un.org/wpp/Download/Standard/Population/). |
|  |  |  | II.i.e At which level of aggregation were the data available? | At the population and gender/age-group level. |
|  | II.ii  Individual Decision Making | | II.ii.a  What are the subjects and objects of the decision-making? On which level of aggregation is decision-making modelled? Are multiple levels of decision making included? | Agent-level decisions:   - Perform a prosocial behavior. This decision depends on the agents’ level of anxiety and religiosity. - Marriage decision. This decision depends on the age, gender, and marital status of the agents.   Marriage-level decisions:   - Reproduction decision. Reproduction is determined by a probability function that incorporates the partners wellbeing and insecurity values. |
|  |  |  | II.ii.b What is the basic rationality behind agent decision-making in the model? Do agents pursue an explicit objective or have other success criteria? | Agents perform a prosocial behavior when the product of their anxiety times their religiosity exceeds a threshold. By performing a prosocial behavior, agents increase the security and religiosity of themselves and that of their neighbors. Agents do not explicitly seek to increase or decrease their insecurity, religiosity, anxiety, etc.  Agents get married in order to have a partner with whom to reproduce.  Reproduction depends on the wellbeing and insecurity levels of the married agents. The higher their levels of wellbeing and insecurity, the higher the probability of reproduction. Wellbeing represents the health status of agents and thus agents with good health status have higher chance of reproduction. Insecurity also increases the probability of reproduction.  Agents do not pursue a success criterion. Success is measure at the population level, by assessing if population increases, decreases, or remains at the same size in time. |
|  |  |  | II.ii.c How do agents make their decisions? | Prosocial behaviors are triggered when the product of anxiety times religion exceeds the *threshold of prosocial behavior*. Anxiety is the product of their insecurity times their sensitivity. Insecurity increases with the number and intensity of threats experienced by agents. All agents experience the same number and intensity of threats. Sensitivity is a variable that remains constant throughout the life span of agents. At initialization, it is determined by drawing values from a normal distribution; when agents are born, it is inherited with error from one of the parents.  Agents start looking for potential partners when they turn 15 y.o. and do so every year till they find a suitable partner. Suitable partners must be single, of the opposite sex, at least 15 y.o., and have a minimum age difference set by user (see reference models). If such a partner is found, agents are set to the state married.  The probability of reproduction is a function of the married agents’ wellbeing and insecurity. The higher their wellbeing and insecurity, the higher their chances of reproduction. |
|  |  |  | II.ii.d Do the agents adapt their behaviour to changing endogenous and exogenous state variables? And if yes, how? | Yes, prosocial behavior is mediated by anxiety and religiosity, and reproduction by wellbeing and insecurity. In turn, these variables are dynamic and mediated by other endogenous variables and exogenous processes.   - Sensitivity is an endogenous static agent variable. Its value is determined at initialization (from a normal distribution) or when agents are born (by inheritance). It remains constant during the lifetime of the agent. - Insecurity is an endogenous dynamic agent variable. It increases with the number and intensity of threats experienced yearly (exogenous processes) and decreases with the performing and receiving prosocial behaviors (endogenous and exogenous processes, respectively). - Anxiety is an endogenous dynamic agent variable. Its value is determined yearly as the product of insecurity times sensitivity. - Religiosity is an endogenous dynamic agent variable. Its value is determined at initialization (from a normal distribution) and when agents are born (by inheritance). It increases with prosocial behaviors performed and received and decreases with time. When agents reach 25 y.o. their value of religiosity remains constant. |
|  |  |  | II.ii.e Do social norms or cultural values play a role in the decision-making process? | No. |
|  |  |  | II.ii.f Do spatial aspects play a role in the decision process? | Yes, the neighbors that are benefited by a prosocial behavior are selected by proximity to the performing agent. First, all agents within a specific radius of distance from the performing agent are selected. If the number of selected agents exceeds the *number of neighbors benefited with prosocial behavior*, benefited agents are selected randomly. |
|  |  |  | II.ii.g Do temporal aspects play a role in the decision process? | Yes – the decrease/increase of religiosity depends on the age of the agent. Once an agent reaches 25 y.o. the level of religiosity remains constant. This may have consequences on the decision of the agent to perform a prosocial behavior. |
|  |  | II.ii.h To which extent and how is uncertainty included in the agents’ decision rules? | | The rate and intensity of threats experience by agents is stochastic. The rate and intensity are governed by a Poisson and exponential distribution, respectively.  The probability of reproduction follows a sigmoidal function governed by the wellbeing and security of the married agents (see submodels).  The probability of dying increases exponentially with lower wellbeing. |
|  | II.iii  Learning | II.iii.a Is individual learning included in the decision process? How do individuals change their decision rules over time as consequence of their experience? | | No, no learning process is included. |
|  |  | II.iii.b Is collective learning implemented in the model? | | No. |
|  | II.iv  Individual Sensing | II.iv.a What endogenous and exogenous state variables are individuals assumed to sense and consider in their decisions? Is the sensing process erroneous? | | Endogenous: agents perceive their religiosity, anxiety, wellbeing, insecurity, and age.  Exogenous processes: agents perceive the threats and their intensity.  The sensing process is not erroneous. |
|  |  | II.iv.b What state variables of which other individuals can an individual perceive? Is the sensing process erroneous? | | Agents perceive the age and gender of other agents (relevant for the marriage process).  The sensing process is not erroneous. |
|  |  | II.iv.c What is the spatial scale of sensing? | | The sensing is Global. |
|  |  | II.iv.d Are the mechanisms by which agents obtain information modelled explicitly, or are individuals simply assumed to know these variables? | | Agents are simply assumed to know these variables. |
|  |  | II.iv.e Are the costs for cognition and the costs for gathering information explicitly included in the model? | | No. |
|  | II.v  Individual Prediction | II.v.a Which data do the agents use to predict future conditions? | | Agents do not make predictions on future conditions. |
|  |  | II.v.b What internal models are agents assumed to use to estimate future conditions or consequences of their decisions? | | N/A. |
|  |  | II.v.c Might agents be erroneous in the prediction process, and how is it implemented? | | N/A. |
|  | II.vi  Interaction | II.vi.a Are interactions among agents and entities assumed as direct or indirect? | | Prosocial behaviors are assumed to be direct. |
|  |  | II.vi.b On what do the interactions depend? | | Agents’ do not interact but benefit others by performing prosocial behaviors. |
|  |  | II.vi.c If the interactions involve communication, how are such communications represented? | | Communication is assumed in the sense that agents know each others gender and age (relevant for the marriage process). |
|  |  | II.vi.d If a coordination network exists, how does it affect the agent behaviour? Is the structure of the network imposed or emergent? | | Neither coordination nor networks are included in the model. |
|  | II.vii  Collectives | II.vii.a Do the individuals form or belong to aggregations that affect and are affected by the individuals? Are these aggregations imposed by the modeller or do they emerge during the simulation? | | N/A. |
|  |  | II.vii.b How are collectives represented? | | At the population level. |
|  | II.viii  Heteroge­neity | II.viii.a Are the agents heterogeneous? If yes, which state variables and/or processes differ between the agents? | | Agents are heterogenous regarding their gender, age, sensitivity, religiosity, wellbeing, anxiety, and insecurity. |
|  |  | II.viii.b Are the agents heterogeneous in their decision-making? If yes, which decision models or decision objects differ between the agents? | | Prosocial behaviors are triggered when the product of anxiety times religiosity exceeds the *threshold of prosocial behavior*.  Probability of reproduction depends on the married agents’ level of wellbeing and insecurity.  Anxiety, religiosity, wellbeing, and insecurity are heterogenous dynamic agents’ variables. |
|  | II.ix  Stochasticity | II.ix.a What processes (including initialisation) are modelled by assuming they are random or partly random? | | Births and deaths are partly random. The probability of giving birth depends on the married agents’ level of wellbeing and insecurity. The probability of dying depends on the wellbeing level of the agent.  The neighbors benefited by a prosocial behavior may be selected at random if the number of neighbors in proximity of the performing agent exceeds the number of neighbors benefited by a prosocial behavior.  Religiosity, sensitivity, and insecurity levels at initialization are also partly random, they are drawn from a normal distribution with given mean and standard deviation.  Threats rate and intensity are also partially random. The number and intensity are drawn from a Poisson and exponential distribution, respectively. |
|  | II.x  Observation | II.x.a What data are collected from the ABM for testing, understanding and analysing it, and how and when are they collected? | | We collect population size and average religiosity of the population every 25 years during a 500 years period. |
|  |  | II.x.b What key results, outputs or characteristics of the model are emerging from the individuals? (Emergence) | | A distinct set of initial conditions may lead to societies that may become extinct, survive, or thrive. |
| 1. Details | III.i  Implementa­tion Details | III.i.a. How has the model been implemented? | | In Anylogic version 8.5.2 |
|  |  | III.i.b Is the model accessible, and if so where? | | It will be placed on a GitHub repository. |
|  | III.ii  Initialisation | III.ii.a What is the initial state of the model world, i.e. at time t=0 of a simulation run? | | At initialization are created 1000 agents. The age values given to these agents are drawn from an age distribution obtained from Norwegian census data and follows a pyramid shape.  The religiosity, insecurity, and sensitivity levels are drawn from a normal distribution with mean and standard deviation indicated by the user.  Initial wellbeing is set according to the agent age (see submodels).  Gender (male or female) is set with a 50-50% chance.  We created a reference model against which we could compare the effects of environmental threats and prosocial behavior in the survival of the society. To create a reference model, we turn off the environmental threats and prosocial behaviors, and search for optimal parameter values (via optimization experiments) that allow a society to keep its population size constant over time. We searched for parameter values related to the reproduction, marriage, wellbeing-age and wellbeing-insecurity processes (see sub-models). The optimal values found where then fixed in the simulations exploring the effects of environmental threats and prosocial behaviors. This allowed us to evaluate the effect of environmental threats and prosocial behaviors in a society that would otherwise remain at a constant population size. |
|  |  | III.ii.b Is the initialisation always the same, or is it allowed to vary among simulations? | | Initialization is always the same. |
|  |  | III.ii.c Are the initial values chosen arbitrarily or based on data? | | The values for the normal distributions related insecurity, anxiety and religiosity were chose arbitrarily.  The age values of the initial population follow census data of the Norwegian population in the 1900’s and have a pyramid shape.  The parameters’ values of the reference models were chosen according to the results of the optimization experiments. |
|  | III.iii Input Data | III.iii.a Does the model use input from external sources such as data files or other models to represent processes that change over time? | | No. |
|  | III.iv Submodels | III.iv.a What, in detail, are the submodels that represent the processes listed in ‘Process overview and scheduling’? | | See equations below. |
|  |  | III.iv.b What are the model parameters, their dimensions and reference values? | | See Tables below. |
|  |  | III.iv.c How were the submodels designed or chosen, and how were they parameterised and then tested? | | See text below. |

# Submodels

***Marriage process***

Once agents reach 15 y.o., they start looking for suitable partners to get married. Suitable partners must be single, of the opposite sex, at least 15 y.o., and have a minimum age difference by the reference models (see reference models below). If such a partner is found, agents are set to the state married and remain so till they die.

***Reproduction process***

The probability of reproduction is given by the following sigmoidal equation (eq. 1):

$Prob.Rep=\frac{1}{1+e^{(-b*\left( x-a \right))}}$ eq 1.

Where *b* represents the parameter *Rep curve shape* (9 in Table 1) and gives the shape of the sigmoidal curve.

*x* is equal to:

$$\frac{(Average.WB.Partners)*Rep.Wei.WB+(Average.Ins.Partners)*Rep.Wei.Ins}{Rep.Wei.WB+Rep.Wei.Ins}$$

representing the importance of WB and insecurity in the reproduction decision (10-11 in Table 1).

*a* is the WB threshold at which reproduction probability is equal to 0.5.

Depending on the values of the reproduction parameters, the probability of reproduction may appear like in fig 4. If agents reproduce, then their WB is decrease by a percentage given by Reproduction Cost. The loss in WB from both partners is then pass into the offspring, and this value becomes the initial WB value of the offspring.

| A) | B) |
| --- | --- |

Figure 1. Reproduction probability for different values of b (colors) and thresholds, 0.5 and 0.1 for A) and B) respectively. Y-axis is reproduction probability and x-axis represents the importance and average values of wellbeing and insecurity of partners (X)

**Newborns process**

When the probability of reproduction is higher than a random uniform number between 0 and 1, a new agent is born. Each married agent contributes a percentage of their wellbeing (cost of reproduction) to the newborn. Thus, newborn agents start their live with a wellbeing value equal to the sum of their parents’ contribution. Further, newborns inherit the religiosity, insecurity, and sensitivity values from one of their parents (this parent is selected at random).

**Wellbeing dynamics**

Wellbeing represents the health of an individual. At initialization, wellbeing values are assigned according to the age of the individual and equation 2 (Fig 2A):

Wellbeing =-0.0000000659*Age^4^ + 0.0000110673*Age^3^ - 0.0006663787*Age^2^ + 0.0159308405*Age + 0.8673401783 (eq. 2)

This polynomial function mimics the survival probability of individuals of both sexes recorded in census data during the 1950’s in Norway (fig 2B). Agents born during the simulation get a wellbeing value equal to the sum of their parents’ contribution. Parents’ contributions is the current wellbeing value of the parents times reproduction cost.

| A) | B) |
| --- | --- |
|  |  |

Figure 2. A) Wellbeing values assigned to agents according to their age at model’s initialization; B) Survival Probability of individuals of both sexes according to age in Norway during the 1950’s.

**Increase/decrease of wellbeing with age**

Every year, the wellbeing of an agent increases with age up to a certain age threshold, where the increase starts becoming lower and lower till it eventually becomes negative, i.e., wellbeing starts to decrease with age (Fig 3). These processes are governed by two different equations, one dictating the increase (eq. 3) and other dictating the decrease (eq. 4).

WB increase equation: 𝐴 ∗ 𝑋^𝐸𝑥𝑝1^ + 𝐶 (eq. 3)

Where:

*C* is the intercept (i.e., the WB increase at age 0)

*A* is a constant equal to - 4 * C

𝑋 is variable standardizing the agent’s age to the age threshold, it is equal to (𝐴𝑔𝑒 −𝑇ℎ𝑟𝑒𝑠ℎ𝑜𝑙𝑑)/(100−𝑇ℎ𝑟𝑒𝑠ℎ𝑜𝑙𝑑)

*Exp1* determines the linearity/non-linearity of the function

WB decrease equation: 𝐴 ∗ 𝑋^𝐸𝑥𝑝2^ + 𝐶 (eq 4.)

Where:

*C* is the intercept (i.e., the WB increase at age 0)

*A* is a constant equal to - 4 * C

*𝑋* is variable standardizing the agent’s age to the age threshold, it is equal to (𝐴𝑔𝑒 −𝑇ℎ𝑟𝑒𝑠ℎ𝑜𝑙𝑑)/(100−𝑇ℎ𝑟𝑒𝑠ℎ𝑜𝑙𝑑)

*Exp2* determines the linearity/non-linearity of the function

Hence, depending on the values of *Exp1* and *Exp2* the increase/decrease of WB with agent may be linear (fig 3A) or non-linear (fig 3B).

| A) | B) |
| --- | --- |
| 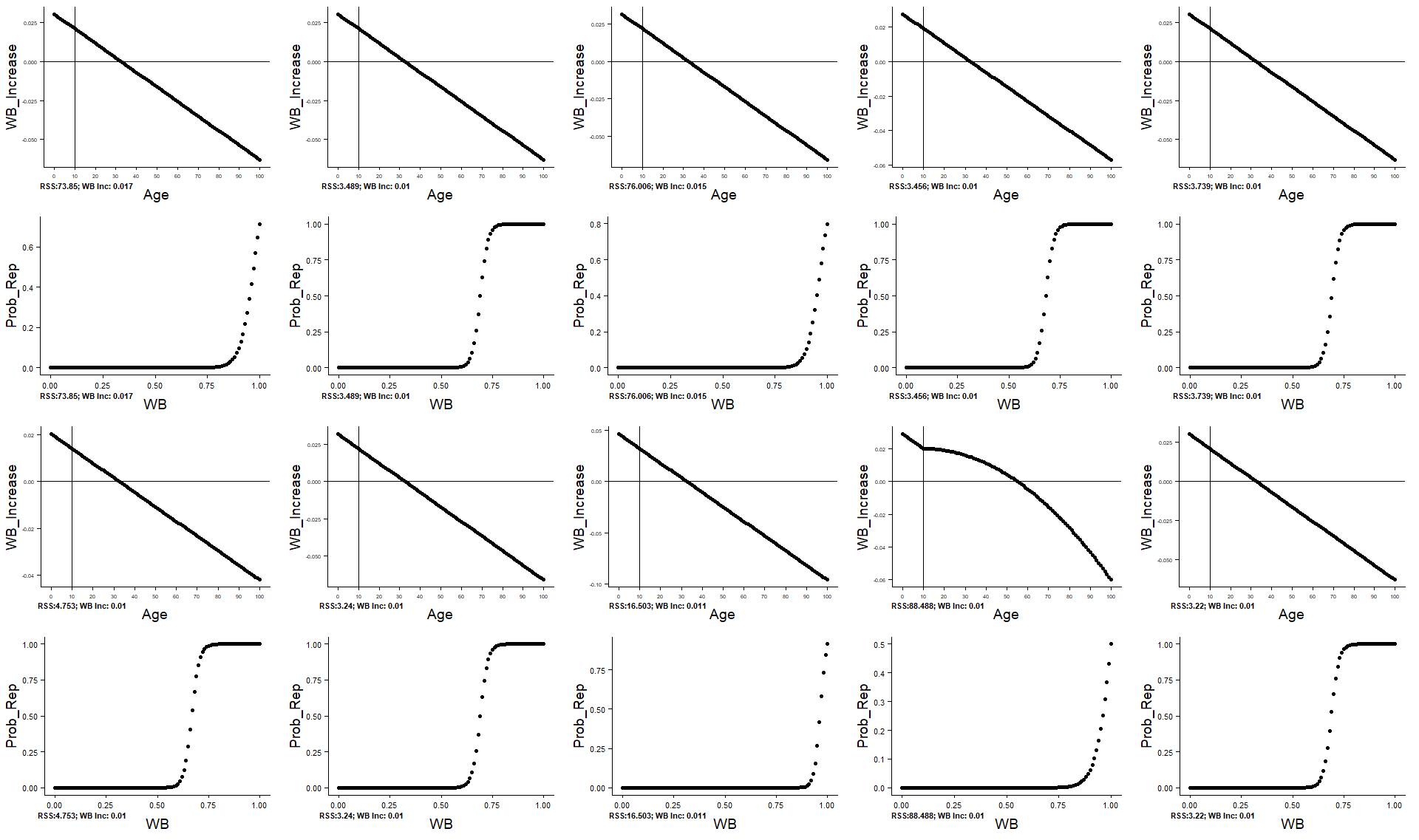 | 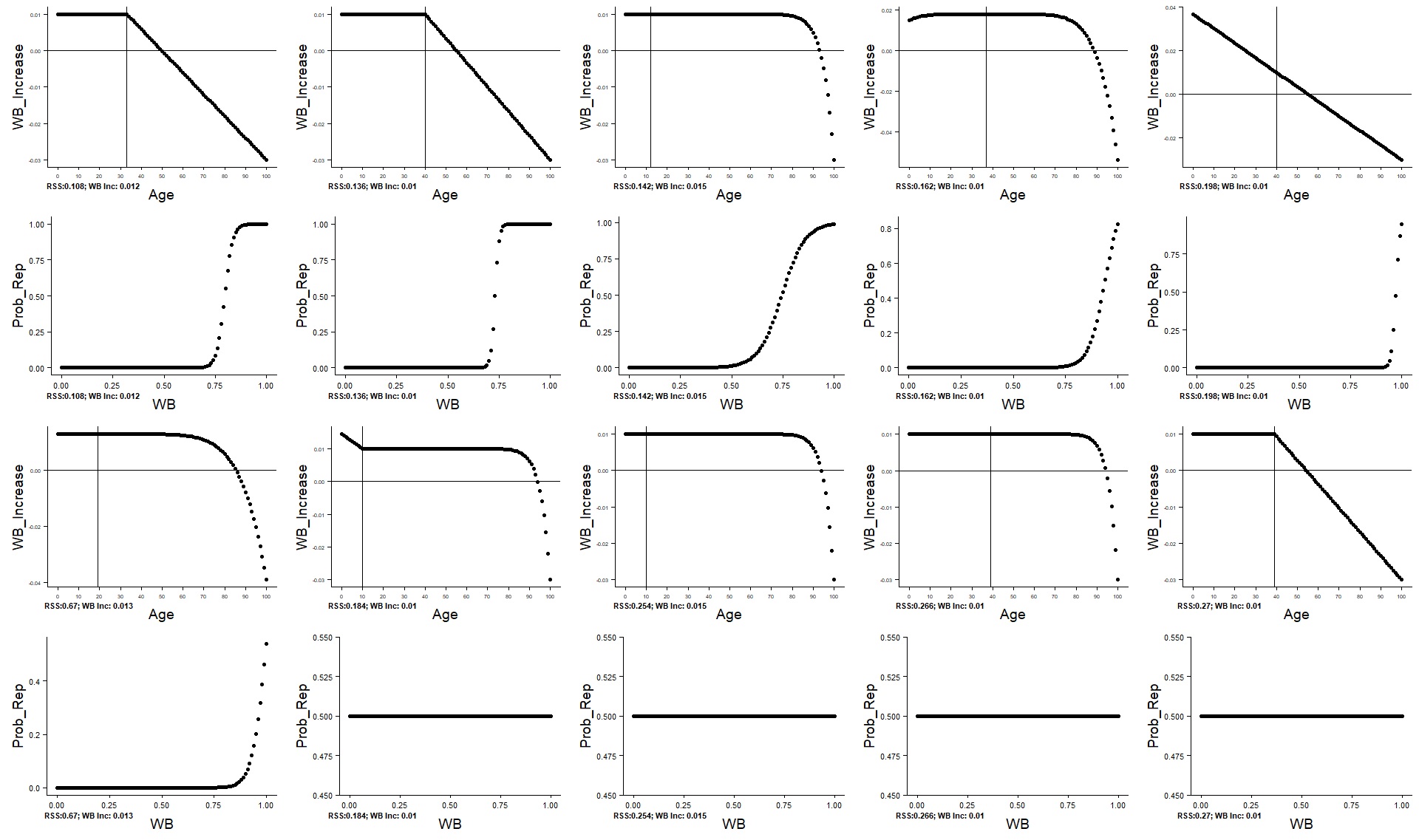 |

Figure 3 Wellbeing increase according to the agent’s age. The vertical line represents the age threshold value and the horizontal line the division between positive (above) and negative (below) values of wellbeing increase.

**Increase/decrease of wellbeing with insecurity**

The agents’ wellbeing also increases and decreases yearly as a function of its level of insecurity. If the level of insecurity in the agent is 0, its wellbeing increases yearly by a max amount. As the level of insecurity increases, the increase in wellbeing becomes lower and lower till it becomes negative, i.e., insecurity becomes detrimental to the wellbeing of the individual. Hence, wellbeing increases when insecurity is low up to a threshold after which wellbeing starts decreasing. This process is governed by two linear equations with different slopes. The first slope is determined by two data points, the first being the max gain in wellbeing when insecurity is 0, and the second being the insecurity threshold value at which the wellbeing gain is 0. The second slope is determined by the insecurity threshold value at which the wellbeing gain is 0, and the max decrease in wellbeing when insecurity is equal to 1 (Fig 4). Note that the maximum increase and maximum decrease in wellbeing, and the insecurity threshold are model’s parameters.


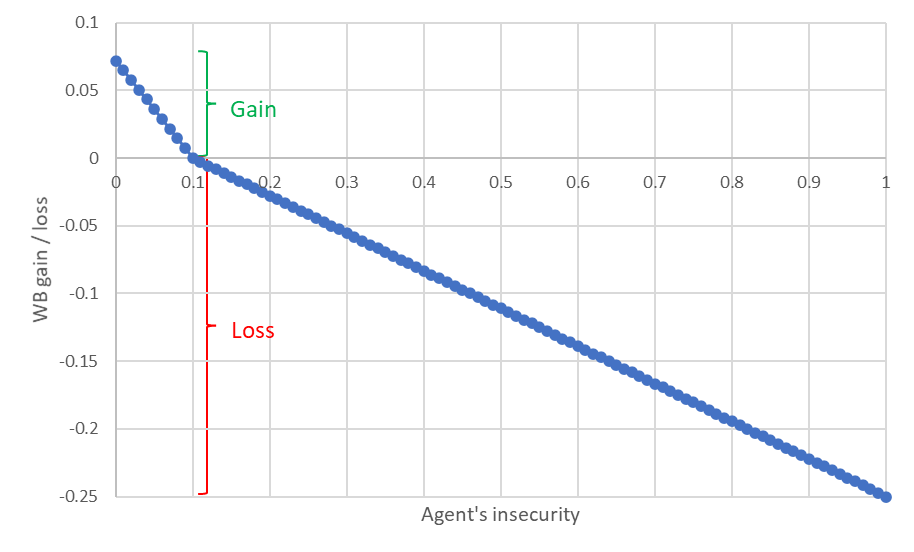


Figure 4 Gain/Loss of WB according to age

The parameters values in this example are age threshold = 16; C = 0.3; Exp1 = 1; Exp = 1. Note that even though the age threshold is 16 yo, WB starts to decrease only when agents become ~40 y.o.

**Mortality process**

Every year agents have a probability of dying according to its current wellbeing value. This probability of dying is a polynomial function that mimics the probability of dying according to age as observed in census data of the Norwegian population in the 1950’s, (equation 5, Fig. 5):

Probability of dying = -4.6343*Age^5^ + 16.042*Age^4^ - 21.98*Age^3^ + 15.041*Age^2^ - 5.237*Age + 0.7719 (eq. 5)

Agents die when their probability of dying in a given year is higher than a random number drawn from a uniform distribution between 0 and 1. Dead agents are removed from the population.

| A) | B) |
| --- | --- |
|  |  |

Figure 5

**Model initialization and processes overview**

The initial population is given by the parameter *Number of Agents* (fixed at 1000). The initial population has a mean religiosity, insecurity, and sensitivity values around 0.5 with a standard deviation of 0.1. The age distribution of agents follows the age distribution of the Norwegian population in the 1900, a pyramid shape. Every year all agents experience a constant environmental threat that increase the insecurity of agents by adding the threat value to the current insecurity value of the agents. If this addition goes above 1, insecurity is set to 1. Next, agents’ anxiety is updated. Anxiety is the product of insecurity times sensitivity, thus the higher the value of insecurity and/or sensitivity, the higher the anxiety of the agents. Then, agents aged 12 y.o. and older are given the chance to perform a prosocial behavior. Prosocial behaviors are triggered when the product of religiosity times anxiety is higher than a threshold (parameter value). If this condition is met, agents perform a prosocial behavior. Prosocial behaviors increase the religiosity and decrease the insecurity of the performing agents and that of a certain number of neighbors. Neighbor agents are considered those agents within a certain radius of distance from the performing agent. If the number of agents in close proximity to the performing agent exceed the number of benefited neighbors (a parameter value); then, the agents that are benefited are selected randomly from those in close proximity. Prosocial behaviors are costly and performing agents reduce their wellbeing by a given parameter value each time they perform a prosocial behavior. After prosocial behaviors are performed, agents increase/decrease their wellbeing according to their current age and insecurity values. Next, female agents that are 15 y.o. or older and that are not married are given the chance to find a partner. Suitable partner are agents that are males, single, above 15 y.o. and with an age difference not higher/lower than a parameter value. If such a partner is found, the marital status of the partners is set to married. Next, female agents that are married and within the age of reproduction [15-49] are given the chance to reproduce. Then, agents that are 25 y.o. or younger, reduce their religiosity values by a percentage given by a parameter value. Finally, agents that die are remove from the population and those that survive start the model cycle again. Figure 6 shows a summary of the model cycle and order of processes during the simulation. The simulation is run for a total of 500 years. Every 25 years we collect the population size and average religiosity of the population.


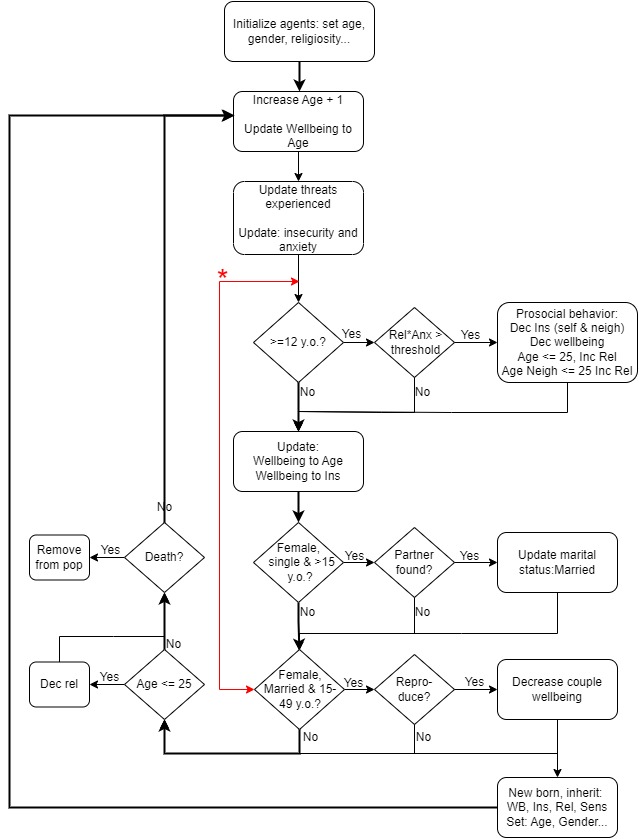


Figure 6 Model cycle and order of processes

*Depending on the setting reproduction may happen (i) before, (ii) after or (iii) randomly before/after prosocial behavior

**Reference model and optimization experiments**

Before exploring the parameter space to investigate the effects of environmental threats and prosocial behavior on population growth, we needed a reference model against which compare these societies. The reference model is needed because otherwise we would not know if societies did not grow because of the effects that threats and prosocial behavior have on the society or because the parameters determining mortality, reproduction and marriage were not accurately calibrated and thus make societies go extinct. Therefore, we performed optimization experiments to find the right combination of parameters’ values (related to the wellbeing, mortality, marriage, and reproduction, Table 1) that produce societies with stable populations when environmental threats and prosocial behavior were turned off.

We used the optimization engine of AnyLogic, which allows the user to obtain a combination of parameter values that increases or decreases a specific output value obtained from an input function. In our case, the input function calculated the residual sum of squares (RSS) between the observed yearly growth rate (i.e., pop_size_y+1_/pop_size_y_) and the expected growth rate if the population size remained constant over time (i.e., 1). The optimization experiments found the combination of parameter values that minimize the output value (RSS). We ran 20 different optimization experiments from which we obtained 20 different combinations of parameters. Each simulation lasted for 500 years. For each combination of parameters, we reran the model 100 times and graph the trajectories of population sizes. Figure 5 shows the trajectories of the population size of the best simulation found via optimization experiments. In these simulations, the population sizes appear slightly growing after 100 years.

| **Parameter** | **Potential Values** | **Description** | **See submodel** |
| --- | --- | --- | --- |
| 1. Reproduction Cost | [1%-50%] | Percentage of wellbeing taken from each parent and pass into offspring | Reproduction  (equation 1) |
| 1. Reproduction mid threshold | [0-1] | Value at which reproduction probability is equal to 0.5 |  |
| 1. Reproduction Curve Shape | [0-100] | Parameters determining the shape of the sigmoidal probability curve |  |
| 1. Intercept value (C) | [0.01-0.3] | Parameters determining the increase / decrease of wellbeing (WB) according to agents’ age | Wellbeing Age dynamics (equations 3 and 4) |
| 1. Age threshold | [10-40] |  |  |
| 1. Exponent increase equation | [1-10] |  |  |
| 1. Exponent decrease equation | [1-10] |  |  |
| 1. Maximum WB gain | [0.01-0.1] | Maximum WB gain when insecurity is 0 | Wellbeing Insecurity dynamics |
| 1. Marriage Age Diff | [2-10] | Maximum age difference between potential partners | Marriage |

Table 1 Parameters optimized to obtain a reference model


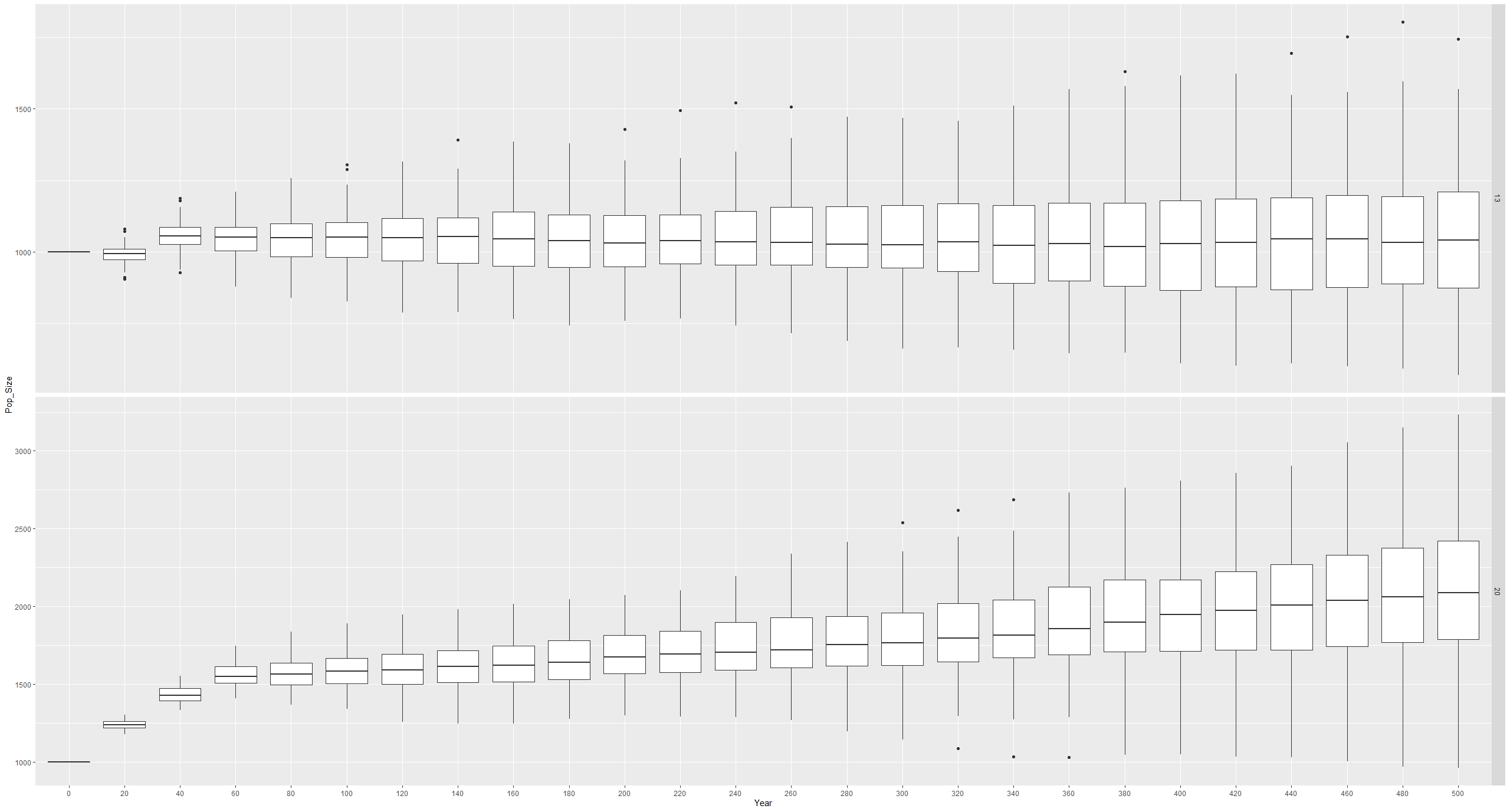


Figure 7 Population size of the best simulation obtained via optimization experiments.

Boxplots represent Q1-Q3 interquartile range, whisker the max-min values, and the line inside the boxplot the median.

The parameters’ values found for the best simulation are shown in table 2.

| Parameter | Sim 13 |
| --- | --- |
| 1. Reproduction Cost | 0.117 |
| 1. Reproduction Threshold | 0.618 |
| 1. Reproduction Curve Shape | 5.324 |
| 1. Intercept value (C) | 0.218 |
| 1. Age threshold | 18 |
| 1. Exponent WB increase equation | 6 |
| 1. Exponent WB decrease equation | 3 |
| 1. Maximum WB gain | 0.062 |
| 1. Marriage Age Diff | 4 |

Table 2

**References**

1. Birgit Müller, Friedrich Bohn, Gunnar Dreßler, Jürgen Groeneveld, Christian Klassert, Romina Martin, Maja Schlüter, Jule Schulze, Hanna Weise, Nina Schwarz. 2013. *Describing human decisions in agent-based models – ODD + D, an extension of the ODD protocol*. Environmental Modelling & Software. Volume 48, Pages 37-48, ISSN 1364-8152, (<https://doi.org/10.1016/j.envsoft.2013.06.003>.)
2. K. Talmont-Kaminski, *Religion as magical ideology: How the supernatural reflects rationality*. Routledge, 2014.
